# Characteristics and Outcomes of Prolonged Venoarterial Extracorporeal Membrane Oxygenation After Cardiac Surgery: The Post-Cardiotomy Extracorporeal Life Support (PELS-1) Cohort Study

**DOI:** 10.1097/CCM.0000000000006349

**Published:** 2024-06-07

**Authors:** Jeroen J. H. Bunge, Silvia Mariani, Christiaan Meuwese, Bas C. T. van Bussel, Michele Di Mauro, Dominik Wiedeman, Diyar Saeed, Matteo Pozzi, Antonio Loforte, Udo Boeken, Robertas Samalavicius, Karl Bounader, Xiaotong Hou, Hergen Buscher, Leonardo Salazar, Bart Meyns, Daniel Herr, Sacha Matteucci, Sandro Sponga, Graeme MacLaren, Claudio Russo, Francesco Formica, Pranya Sakiyalak, Antonio Fiore, Daniele Camboni, Giuseppe Maria Raffa, Rodrigo Diaz, I-wen Wang, Jae-Seung Jung, Jan Belohlavek, Vin Pellegrino, Giacomo Bianchi, Matteo Pettinari, Alessandro Barbone, José P. Garcia, Kiran Shekar, Glenn J. R. Whitman, Diederik Gommers, Dinis Dos Reis Miranda, Roberto Lorusso

**Affiliations:** 1 Department of Intensive Care Adults, Erasmus MC, Rotterdam, The Netherlands.; 2 Deparment of Cardiology, Thoraxcenter, Erasmus MC, Rotterdam, The Netherlands.; 3 Cardio-Thoracic Surgery Department and Cardiovascular Research Institute Maastricht, Maastricht, The Netherlands.; 4 Department of Intensive Care Medicine, Cardiovascular Research Institute Maastricht (CARIM), Maastricht, The Netherlands.; 5 Department of Cardiology, Pierangeli Hospital, Pescara, Italy.; 6 Department of Cardiac Surgery, Medical University of Vienna, Vienna, Austria.; 7 Department of Cardiac Surgery, University Hospital St. Pölten, St. Pölten, Austria.; 8 Department of Cardiac Surgery, Leipzig Heart Center, Leipzig, Germany.; 9 Department of Cardiac Surgery, Louis Pradel Cardiologic Hospital, Lyon, France.; 10 Division of Cardiac Surgery, IRCCS Azienda Ospedaliero-Universitaria di Bologna, Bologna, Italy.; 11 Deparment of Surgical Sciences, University of Turin, Turin, Italy.; 12 Department of Cardiac Surgery, Medical Faculty, Heinrich Heine University, Duesseldorf, Germany.; 13 II Department of Anesthesiology, Centre of Anesthesia, Intensive Care and Pain Management, Vilnius University Hospital Santariskiu Klinikos, Vilnius, Lithuania.; 14 Division of Cardiothoracic and Vascular Surgery, Pontchaillou University Hospital, Rennes, France.; 15 Center for Cardiac Intensive Care, Beijing Institute of Heart, Lung, and Blood Vessels Diseases, Beijing Anzhen Hospital, Capital Medical University, Beijing, China.; 16 Department of Intensive Care Medicine, Center of Applied Medical Research, St Vincent’s Hospital, University of New South Wales, Sydney, NSW, Australia.; 17 Department of Cardiology, Fundación Cardiovascular de Colombia, Bucaramanga, Colombia.; 18 Department of Cardiac Surgery, University Hospitals Leuven and Department of Cardiovascular Sciences, University of Leuven, Leuven, Belgium.; 19 Departments of Medicine and Surgery, University of Maryland, Baltimore, MD.; 20 SOD Cardiochirurgia Ospedali Riuniti “Umberto I-Lancisi-Salesi” Università Politecnica delle Marche, Ancona, Italy.; 21 Division of Cardiac Surgery, Cardiothoracic Department, University Hospital of Udine, Udine, Italy.; 22 Cardiothoracic Intensive Care Unit, National University Heart Centre, National University Hospital, Singapore, Singapore.; 23 Cardiac Surgery Unit, Cardiac Thoracic and Vascular Department, Niguarda Hospital, Milan, Italy.; 24 Department of Medicine and Surgery, Cardiac Surgery Clinic, San Gerardo Hospital, University of Milano-Bicocca, Monza, Italy.; 25 Department of Medicine and Surgery, University of Parma, Parma, Italy.; 26 Cardiac Surgery Unit, University Hospital of Parma, Parma, Italy.; 27 Division of Cardiovascular and Thoracic Surgery, Department of Surgery, Faculty of Medicine Siriraj Hospital, Mahidol University, Bangkok, Thailand.; 28 Department of Cardio-Thoracic Surgery, University Hospital Henri-Mondor, Créteil, Paris, France.; 29 Department of Cardiothoracic Surgery, University Medical Center Regensburg, Regensburg, Germany.; 30 Department for the Treatment and Study of Cardiothoracic Diseases and Cardiothoracic Transplantation, IRCCS-ISMETT (Istituto Mediterraneo per i Trapianti e Terapie ad Alta Specializzazione), Palermo, Italy.; 31 ECMO Unit, Centro Cardiovascular Red Salud Santiago and Hospital San Juan de Dios, Santiago, Chile.; 32 Division of Cardiac Surgery, Memorial Healthcare System, Hollywood, FL.; 33 Department of Thoracic and Cardiovascular Surgery, Korea University Anam Hospital, Seoul, South Korea.; 34 2nd Department of Internal Medicine, Cardiovascular Medicine General Teaching Hospital and 1st Faculty of Medicine, Charles University in Prague, Prague, Czech Republic.; 35 Intensive Care Unit, The Alfred Hospital, Melbourne, VIC, Australia.; 36 Ospedale del Cuore Fondazione Toscana “G. Monasterio,” Massa, Italy.; 37 Department of Cardiovascular Surgery, Ziekenhuis Oost-Limburg, Genk, Belgium.; 38 Cardiac Surgery Unit, IRCCS Humanitas Research Hospital, Rozzano, Milan, Italy.; 39 IU Health Advanced Heart & Lung Care, Indiana University Methodist Hospital, Indianapolis, IN.; 40 Adult Intensive Care Services, The Prince Charles Hospital, Brisbane, QLD, Australia.; 41 Cardiac ICU, Johns Hopkins Hospital, Baltimore, MD.

**Keywords:** acute heart failure, cardiac surgery, extracorporeal membrane oxygenation, mechanical circulatory support, post-cardiotomy cardiogenic shock

## Abstract

**OBJECTIVES:**

Most post-cardiotomy (PC) extracorporeal membrane oxygenation (ECMO) runs last less than 7 days. Studies on the outcomes of longer runs have provided conflicting results. This study investigates patient characteristics and short- and long-term outcomes in relation to PC ECMO duration, with a focus on prolonged (> 7 d) ECMO.

**DESIGN:**

Retrospective observational cohort study.

**SETTING:**

Thirty-four centers from 16 countries between January 2000 and December 2020.

**PATIENTS:**

Adults requiring post PC ECMO between 2000 and 2020.

**INTERVENTIONS:**

None.

**MEASUREMENTS AND MAIN RESULTS:**

Characteristics, in-hospital, and post-discharge outcomes were compared among patients categorized by ECMO duration. Survivors and nonsurvivors were compared in the subgroup of patients with ECMO duration greater than 7 days. The primary outcome was in-hospital mortality. Two thousand twenty-one patients were included who required PC ECMO for 0–3 days (*n* = 649 [32.1%]), 4–7 days (*n* = 776 [38.3%]), 8–10 days (*n* = 263 [13.0%]), and greater than 10 days (*n* = 333 [16.5%]). There were no major differences in the investigated preoperative and procedural characteristics among ECMO duration groups. However, the longer ECMO duration category was associated with multiple complications including bleeding, acute kidney injury, arrhythmias, and sepsis. Hospital mortality followed a U-shape curve, with lowest mortality in patients with ECMO duration of 4–7 days (*n* = 394, 50.8%) and highest in patients with greater than 10 days ECMO support (*n* = 242, 72.7%). There was no significant difference in post-discharge survival between ECMO duration groups. In patients with ECMO duration greater than 7 days, age, comorbidities, valvular diseases, and complex procedures were associated with nonsurvival.

**CONCLUSIONS:**

Nearly 30% of PC ECMO patients were supported for greater than 7 days. In-hospital mortality increased after 7 days of support, especially in patients undergoing valvular and complex surgery, or who had complications, although the long-term post-discharge prognosis was comparable to PC ECMO patients with shorter support duration.

**KEY POINTS**

**Question:** To investigate outcomes after longer duration of post-cardiotomy extracorporeal membrane oxygenation (ECMO).

**Findings:** In this large multicenter study, we found a U-shape curve of mortality and ECMO duration. Longer ECMO duration (> 7 d) is associated with complications and increased mortality, but post-discharge survival is good. Age, comorbidities, and complications like acute kidney injury and multiple organ failure were associated with mortality after longer ECMO support.

**Meaning:** Although this study helps in identifying patients with reasonable chances for successful outcomes after longer ECMO support, the decision to continue or cease post-cardiotomy ECMO is complex: multidisciplinary teams should take comorbidities, complications, and patient values into account.

Venoarterial extracorporeal membrane oxygenation (ECMO) has increasingly been used for post-cardiotomy (PC) shock during the last decades (1–3). PC ECMO is used to overcome a period of cardiac dysfunction, which may be caused (among others) by long cross-clamp times, inadequate myocardial protection, perioperative myocardial ischemia/infarction, or arrhythmias and which may be enhanced by poor preoperative cardiac function or emergency surgery.

Typically, PC ECMO is used for 3–7 days (4–6). The first 24–48 hours may be necessary to overcome acute instability and metabolic derangements (5). The consecutive days allow for further cardiac and other organ recovery, while aiming for a negative fluid balance and overcoming postoperative systemic inflammation (7). As soon as there are signs of cardiac recovery after the acute phase, ECMO weaning attempts should be performed (8). The timeframe for recovery and consecutive successful decannulation is variable and hard to predict. Historically, 3–7 days have been advised, but these numbers seem largely based on clinical experience and observational studies (4–6), and longer ECMO runs could favor patients. Indeed, the duration of eventual cardiac recovery may depend on the mechanism of injury (i.e., stunning or myocardial infarction) and clinical phenotype (i.e., predominantly right ventricular [RV] failure or left ventricular [LV]/biventricular failure). Furthermore, rethoracotomy for bleeding, other invasive procedures, tachyarrhythmias, or infections, may act as an additional insult for recovering patients and may delay successful weaning.

Evidence on duration of PC ECMO runs is unclear, although some studies showed that longer PC ECMO runs have been associated with increased mortality (9–12); with major variation in the effect sizes of ECMO duration between studies. In contrast, however, ECMO duration has not been identified as an independent determinant of mortality in a large meta-regression analysis (11). Nevertheless, the duration of ECMO support is often recognized as a risk factor for ECMO-related complications, such as infection, bleeding, and neurologic events (13–15), which, in turn, affects PC ECMO duration. Observational studies focusing on identifying patients with reasonable chances for successful outcomes after longer PC ECMO runs are lacking.

We investigate patients within the Post-Cardiotomy Extracorporeal Life Support (PELS-1) study, which is a large retrospective multicenter study on PC ECMO (16). In the present investigation we aim to characterize patients and their clinical outcomes depending on PC ECMO duration, with a focus on those who underwent ECMO support for more than 7 days, and we hypothesized that patients with PC ECMO runs longer than 7 days can have favorable outcomes.

## MATERIALS AND METHODS

### Study Design

The PELS-1 is a retrospective, multicenter, observational study (ClinicalTrials.gov: NCT03857217), which enrolled adult patients (≥ 18 yr) supported by ECMO after cardiac surgery in 34 centers from 16 countries between January 2000 and December 2020 and has been described extensively (16–19). All PELS-1 investigations are conducted in accordance with institutional ethical standards and the declaration of Helsinki (1975). Primary institutional review board (IRB) approval was obtained through the medical ethical review committee of the leading center (Maastricht University Medical Centre+, Maastricht, The Netherlands, IRB-approval METC-2018-0788, February 27, 2019, Post-Cardiotomy Extra-Corporeal Life Support Study [PELS]). Need for informed consent was waived based on the retrospective nature of the study, the emergency of the performed procedures, and the pseudonymization of shared data. IRB approval was obtained in all centers based on the leading center’s protocol.

### Study Population

For this study, all adult patients included in the PELS-1 study undergoing PC ECMO and with a known ECMO support duration were analyzed. Exclusion criteria were unknown mortality status and extracorporeal life support other than venoarterial ECMO, ECMO after discharge or before surgery, ECMO after noncardiac surgical procedures, and ECMO implantation not strictly related to cardiac surgery hospitalization.

### Data Collection and Outcomes

Data were collected, by local investigators for each participating hospital, using a dedicated electronic case report form (data.castoredc.com, Castor, Amsterdam, The Netherlands), according to predefined variable and outcome definitions (**Supplementary Table 1**, http://links.lww.com/CCM/H558). The coordinating center centrally managed the full dataset. The primary outcome of interest for the current study was in-hospital mortality, with two more specific definitions: on ECMO mortality (if the patient died while on PC ECMO support) and post-weaning mortality (if the patient died after decannulation but before hospital discharge). Secondary outcomes were hospital length of stay, in-hospital complications, postoperative procedures (percutaneous coronary intervention, cardiac surgery, and vascular surgery), cause of in-hospital death, and long-term survival status (definitions in Supplementary Table 1, http://links.lww.com/CCM/H558).

### Statistical Analysis

Patients were stratified in four groups based on PC ECMO support duration (0–3, 4–7, 8–10, > 10 d). This was based on previous studies, the size of the cohort determining strata and clinical reasoning (7, 9, 10). Their demographic and clinical variables were expressed as numbers (valid percentage on available data, excluding missing values) for categorical variables and median (interquartile range) or mean and sd for continuous variables. All descriptive statistics were performed on available original data after missing value analysis (**Supplementary Table 2**, http://links.lww.com/CCM/H558). No imputations were performed. Categorical data were compared using chi-square test. Continuous variables were analyzed using the Kruskal-Wallis test. In case of significant differences between the four ECMO duration groups, post hoc comparisons were performed and adjusted by the Bonferroni correction for multiple tests. Stacked bar plots were designed to represent the distributions of levels within each categorical variable and compare them between study groups. Crude survival analysis was performed using Kaplan-Meier curves, and differences in survival were assessed with the log-rank test. Based on the possible variations in ECMO management over the study period, we performed a sensitivity analysis excluding patients who received PC ECMO between 2000 and 2010 (20).

Finally, further descriptive subgroup analyses were performed to compare survivors and nonsurvivors in patients with PC ECMO run greater than 7 days (long PC ECMO duration group) based on previous studies that have shown greater mortality in this group compared with shorter PC ECMO runs (9). We considered a two-sided *p* value of less than 0.05 statistically significant. All data were merged from de-identified files into SPSS 26.0 (IBM, Armonk, NY), and R 4.1.2 (R Foundation for Statistical Computing, Vienna, Austria) for data management and statistical analysis.

## RESULTS

### Patients, Procedural, and PC ECMO Characteristics

In total, 2163 patients were included in the PELS-1 study. For the present study, 2021 patients had complete data to be analyzed (**Supplementary Fig. 1**, http://links.lww.com/CCM/H558), after exclusion of patients who missed primary outcome data (*n* = 72), who underwent venovenous ECMO (*n* = 33), or who missed data on PC ECMO duration (*n* = 37). Most centers were heart transplantation centers (**Supplementary Table 3**, http://links.lww.com/CCM/H558). Patients were stratified in four groups based on PC ECMO duration: 0–3 days (*n* = 649 [32.1%]), 4–7 days (*n* = 776 [38.3%]), 8–10 days (*n* = 263 [13.0%]), and greater than 10 days (*n* = 333 [16.5%]). As shown in **Table 1**; and **Supplementary Table 4** (http://links.lww.com/CCM/H558), groups differed, among others, with respect to age (> 10 d of ECMO support: 63 yr [53–71 yr] and 4–7 d group: 66 yr [65–73 yr]; *p* = 0.016) and LV ejection fraction was (LVEF) (45% [30–45%] in > 10 d group vs. 50% [30–60%] in 0–3 d group; *p* = 0.033).

**TABLE 1.** Preoperative Characteristics of the Overall Population Stratified According to Post-Cardiotomy Extracorporeal Membrane Oxygenation Duration

Baseline Variables	0–3 d (*n* = 649)	4–7 d (*n* = 776)	8–10 d (*n* = 263)	> 10 d (*n* = 333)	*p*
Demographics					
Age (yr)	65 (56–72)	66 (56–73)	64 (55–71)	63 (53–71)	0.016
Female	266 (41.0)	324 (41.8)	100 (38.0)	139 (41.7)	0.742
Body mass index (kg/m^2^)	26.5 (23.6–30.1)	26.0 (23.5–29.4)	27.2 (24.0–30.6)	26.8 (23.8–30.7)	0.058
Comorbidities					
Hypertension	422 (68.0)	486 (64.1)	171 (69.2)	208 (64.4)	0.287
Diabetes mellitus	157 (24.2)	186 (24.0)	87 (33.1)	86 (25.8)	0.023
Smoking	138 (25.3)	189 (28.9)	62 (28.2)	74 (25.4)	0.478
Chronic obstructive pulmonary disease	68 (10.9)	73 (9.9)	28 (11.0)	36 (11.3)	0.884
Dialysis	49 (7.9)	65 (8.6)	27 (10.6)	34 (10.5)	0.430
Atrial fibrillation	160 (24.7)	225 (29.0)	71 (27.0)	74 (22.3)	0.084
Previous MI	160 (24.7)	219 (28.2)	66 (25.1)	103 (30.9)	0.141
Recent MI (< 30 d)	72 (11.6)	89 (11.7)	25 (10.1)	44 (13.6)	0.630
Previous percutaneous coronary intervention	120 (18.6)	125 (16.3)	40 (15.3)	59 (17.8)	0.547
Previous stroke	96 (14.8)	105 (13.5)	45 (17.1)	33 (9.9)	0.065
Previous transient ischemic attack	16 (2.9)	15 (2.2)	3 (1.3)	5 (1.6)	0.467
Pulmonary hypertension (> 50 mm Hg)	110 (17.1)	184 (23.8)	61 (23.3)	69 (20.9)	0.017
Prior cardiac surgery	156 (24.0)	212 (27.3)	74 (28.1)	87 (26.1)	0.460
Creatinine (µmol/L)	102 (80–141)	101 (80–140)	100 (79–133)	102 (80–149)	0.750
Preoperative status					
Left ventricular ejection fraction (%)	50 (30–60)	45 (30–60)	45 (30–60)	45 (30–55)	0.033
New York Heart Association class					0.368
Class I	50 (8.2)	51 (6.9)	15 (6.0)	25 (7.7)	
Class II	123 (20.1)	151 (20.5)	51 (20.2)	87 (26.8)	
Class III	238 (39.0)	304 (41.4)	99 (39.3)	117 (36.0)	
Class IV	200 (32.7)	229 (31.2)	87 (34.5)	96 (29.5)	
European System for Cardiac Operative Risk Evaluation II	6.84 (2.9–18.9)	7.34 (3.1–17.6)	8.70 (2.6–20.1)	9.43 (3.2–19.3)	0.608
Cardiogenic shock	135 (21.1)	150 (19.7)	61 (23.5)	83 (25.1)	0.206
Intubation	80 (12.3)	78 (10.1)	33 (12.5)	39 (11.7)	0.513
Cardiac arrest	74 (11.5)	60 (7.8)	23 (8.8)	29 (8.8)	0.119
Preoperative intra-aortic balloon pump	47 (7.3)	79 (10.2)	30 (11.4)	33 (9.9)	0.140
Right ventricular failure	61 (10.8)	66 (9.8)	21 (9.4)	33 (10.9)	0.877
Urgent surgery	159 (24.8)	171 (22.3)	52 (19.8)	63 (19.0)	0.157
Emergency surgery	166 (25.9)	191 (25.0)	78 (29.8)	84 (25.5)	0.495

MI = myocardial infarction.

Data are reported as *n* (% as valid percentage excluding missing values), mean ± sd, or median (interquartile range). *p* values by χ^2^ test (for categorical data) or Kruskal-Wallis test (for continuous data) indicate statistically significant differences between post-cardiotomy extracorporeal membrane oxygenation duration groups.

Regarding procedural characteristics (**Table 2**; and Supplementary Table 4, http://links.lww.com/CCM/H558), there were differences in the distribution of heart transplantation (*p* = 0.039), aortic valve surgery (*p* = 0.046), and mitral valve surgery (*p* = 0.029) among PC ECMO duration groups. However, these characteristics did not show a clear increase or decrease across the groups of PC ECMO duration. There were no statistically significant differences in terms of indication for PC ECMO, implant timing and cannulation approach (**Table 3**).

**TABLE 2.** Procedural Characteristics Stratified According to Post-Cardiotomy Extracorporeal Membrane Oxygenation Duration

Operative Variables	0–3 d (*n* = 649)	4–7 d (*n* = 776)	8–10 d (*n* = 263)	> 10 d (*n* = 333)	*p*
Weight of surgery					0.605
Unknown	4 (0.6)	4 (0.5)	1 (0.4)	4 (1.2)	
Isolated CABG	106 (16.3)	140 (18.0)	49 (18.6)	70 (21.0)	
Isolated non-CABG	383 (59.0)	431 (55.5)	144 (54.8)	174 (52.3)	
Two procedures	44 (6.8)	57 (7.3)	25 (9.5)	21 (6.3)	
Three or more procedures	112 (17.3)	144 (18.6)	44 (16.7)	64 (19.2)	
Surgical procedures					
CABG	277 (42.7)	341 (43.9)	126 (47.9)	153 (45.9)	0.480
Aortic valve surgery	235 (36.2)	246 (31.7)	107 (40.7)	113 (33.9)	0.046
Mitral valve surgery	178 (27.4)	269 (34.7)	84 (31.9)	99 (29.8)	0.029
Tricuspid valve surgery	74 (11.4)	114 (14.7)	38 (14.4)	43 (12.9)	0.299
Aortic surgery	142 (21.9)	133 (17.1)	46 (17.5)	54 (16.2)	0.066
Pulmonary valve surgery	5 (0.8)	4 (0.5)	2 (0.8)	1 (0.3)	0.793
Left ventricular assist device	10 (1.5)	6 (0.8)	1 (0.4)	6 (1.8)	0.212
Right ventricular assist device	1 (0.2)	1 (0.1)	1 (0.4)	3 (0.9)	0.148
Atrial septal defect repair	8 (1.2)	18 (2.3)	2 (0.8)	10 (3.0)	0.096
Ventricular septal defect repair	21 (3.2)	20 (2.6)	11 (4.2)	16 (4.8)	0.243
Ventricular surgery	25 (3.9)	28 (3.6)	9 (3.4)	13 (3.9)	0.984
Rhythm surgery	17 (2.6)	29 (3.7)	5 (1.9)	14 (4.2)	0.270
Pulmonary embolectomy	4 (0.6)	10 (1.3)	4 (1.5)	4 (1.2)	0.546
Pulmonary endarterectomy	6 (0.9)	21 (2.7)	5 (1.9)	14 (4.2)	0.546
Heart transplantation	65 (10.0)	94 (12.1)	17 (6.5)	28 (8.4)	0.039
Procedural characteristics					
Off-pump surgery	30 (4.7)	28 (3.7)	16 (6.1)	8 (2.4)	0.115
Conversion to CPB	11 (36.7)	9 (29.0)	3 (18.8)	2 (25.0)	0.634
CPB time (min)	195 (132–290)	206 (145–286)	199 (145–280)	218 (138–302)	0.358
Cross clamp time (min)	93 (62–143)	101 (67–146)	104 (67–148)	103 (63–165)	0.173
Intraoperative lactate (mmol/L)	5.3 (2.8–10.0)	5.6 (2.6–8.6)	5.0 (2.4–7.4)	5.0 (2.8–7.8)	0.266

CABG = coronary artery bypass graft, CPB = cardiopulmonary bypass.

Data are reported as *n* (% as valid percentage excluding missing values) or median (interquartile range). *p* values by χ^2^ test (for categorical data) or Kruskal-Wallis test (for nonparametric continuous data) indicate statistically significant differences between groups.

**TABLE 3.** Details on Extracorporeal Membrane Oxygenation Stratified According Extracorporeal Membrane Oxygenation Duration

ECMO Variables	0–3 d (*n* = 649)	4–7 d (*n* = 776)	8–10 d (*n* = 263)	> 10 d (*n* = 333)	*p*
Post-cardiotomy ECMO indication					0.171
Failure to wean	260 (40.9)	308 (40.3)	92 (35.4)	114 (35.3)	
Acute pulmonary embolism	1 (0.2)	1 (0.1)	0 (0.0)	1 (0.3)	
Arrhythmia	14 (2.2)	16 (2.1)	7 (2.7)	6 (1.9)	
Cardiac arrest	75 (11.8)	50 (6.5)	20 (7.7)	23 (7.1)	
Cardiogenic shock	148 (23.3)	191 (25.0)	71 (27.3)	94 (29.1)	
Pulmonary hemorrhage	2 (0.3)	5 (0.7)	0 (0.0)	1 (0.3)	
Right ventricular failure	63 (9.9)	94 (12.3)	31 (11.9)	47 (14.6)	
Respiratory failure	21 (3.3)	24 (3.1)	13 (5.0)	13 (4.0)	
Biventricular failure	40 (6.3)	65 (8.5)	21 (8.1)	22 (6.8)	
Other	11 (1.7)	11 (1.4)	5 (1.9)	2 (0.6)	
Chest status					0.334
Chest closed	282 (60.5)	316 (55.3)	119 (59.2)	129 (55.6)	
Chest open	184 (39.5)	255 (44.7)	82 (40.8)	103 (44.4)	
Cannulation approach					0.102
Only central cannulation	112 (17.3)	120 (15.5)	42 (16.0)	65 (19.5)	
Only peripheral cannulation	322 (49.6)	367 (47.3)	117 (44.5)	152 (45.6)	
Mixed/switch cannulation	205 (31.6)	273 (35.2)	103 (39.2)	115 (34.5)	
Unknown	10 (1.5)	16 (2.1)	1 (0.4)	1 (0.3)	
ECMO implant timing					0.130
Intraoperative	425 (65.5)	488 (62.9)	152 (57.8)	201 (60.4)	
Postoperative	224 (34.5)	288 (37.1)	111 (42.2)	132 (39.6)	
Left ventricular venting	150 (28.0)	189 (29.9)	71 (34.6)	104 (35.7)	0.072

ECMO = extracorporeal membrane oxygenation.

Data are reported as *n* (%) as valid percentage excluding missing values. *p* values by χ^2^ test indicate statistically significant differences between post-cardiotomy ECMO duration groups.

### Outcomes

**Figure 1.**
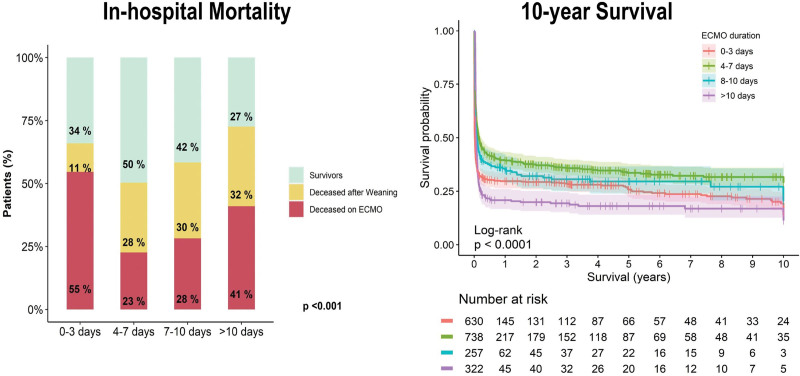
Short and long-term outcomes of post-cardiotomy (PC) extracorporeal membrane oxygenation (ECMO) according to ECMO duration groups. **A**, *Stacked bar plots* representing mortality while on PC ECMO, in-hospital mortality after PC ECMO explantation, and hospital survival by PC ECMO duration groups. **B**, Kaplan-Meier survival curves with 95% CIs for the various PC ECMO duration groups, starting at time of ECMO implantation.

**Figure 2.**
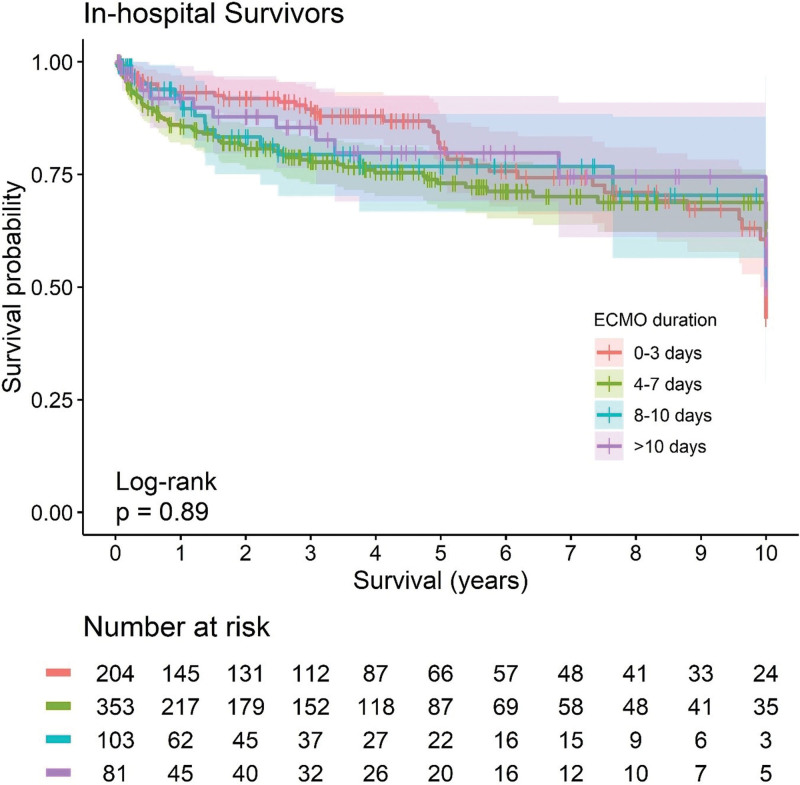
Kaplan-Meier survival curves with 95% CIs for the various post-cardiotomy extracorporeal membrane oxygenation (ECMO) duration groups. Long-term survival of hospital survivors.

Complications differed between groups (**Table 4**; and Supplementary Table 4, http://links.lww.com/CCM/H558), especially in terms of bleeding (*p* ≤ 0.001), arrhythmias (*p* < 0.001), leg ischemia (*p* = 0.030), bowel ischemia (*p* = 0.003), abdominal surgery (*p* = 0.015), acute kidney injury (AKI), pneumonia, septic shock, cardiac surgery, and multiple organ failure (all *p* < 0.001). Occurrence of bleeding, arrhythmias, AKI, and septic shock showed a gradual increase in relation to ECMO duration. In-hospital mortality was 60.3% (*n* = 1219) in the overall cohort and differed significantly between groups, with the lowest mortality in the 4–7 days group (*n* = 394, 50.8%) and the highest one in greater than 10 days group (*n* = 242, 72.7%; *p* < 0.001). Timing of in-hospital death (on ECMO or after weaning) was also different for the various groups (*p* < 0.001; **Fig. 1**; and **Supplementary Fig. 2**, http://links.lww.com/CCM/H558). There were differences in cause of death as well, with more multiple organ failure and sepsis in PC ECMO duration 7–10 and greater than 10 days, with the result for less sepsis in patients dying after short (0–3 d) PC ECMO duration being statistically significant after post hoc comparisons between the individual ECMO duration groups (Supplementary Table 4, http://links.lww.com/CCM/H558). In contrast, bleeding was more often listed as cause of death in the 0–3 days PC ECMO group (Table 4; *p* < 0.001). Overall survival was mainly affected by differences between groups in terms of early mortality (Fig. 1), but long-term survival after hospital discharge was similar for all groups (**Fig. 2**).

**TABLE 4.** Postoperative Outcomes Stratified According to Post-Cardiotomy Extracorporeal Membrane Oxygenation Duration

Outcomes	0–3 d (*n* = 649)	4–7 d (*n* = 776)	8–10 d (*n* = 263)	> 10 d (*n* = 333)	*p*
ICU stay (d)	4 (2–13)	15 (8–26)	19 (10–35)	22 (14–38)	< 0.001
Hospital stay (d)	7 (2–27)	25 (10–44)	26 (11–45)	24 (16–47)	< 0.001
Postoperative bleeding	316 (49.5)	434 (56.9)	167 (63.7)	228 (69.1)	< 0.001
Requiring rethoracotomy	199 (32.8)	283 (39.2)	120 (47.8)	155 (49.1)	< 0.001
Cannulation site bleeding	60 (9.4)	90 (11.8)	40 (15.3)	55 (16.7)	0.004
Diffuse no surgical-related bleeding	147 (25.0)	176 (25.1)	67 (28.4)	75 (24.8)	0.738
Cerebral hemorrhage	14 (2.3)	33 (4.5)	7 (2.9)	12 (3.7)	0.174
Stroke	59 (9.2)	82 (10.6)	31 (11.9)	42 (12.6)	0.358
Arrhythmia	151 (25.5)	245 (34.4)	90 (38.3)	135 (43.0)	< 0.001
Leg ischemia	52 (8.6)	81 (10.9)	21 (8.6)	46 (14.5)	0.030
Cardiac arrest	108 (18.2)	108 (15.1)	38 (16.2)	44 (14.0)	0.313
Bowel ischemia	19 (3.2)	45 (6.3)	22 (9.4)	21 (6.7)	0.003
Right ventricular failure	110 (18.9)	133 (19.1)	68 (29.3)	73 (23.9)	0.003
Acute kidney injury	277 (46.9)	424 (59.3)	148 (63.0)	210 (68.0)	< 0.001
Pneumonia	69 (11.9)	182 (26.1)	70 (30.0)	88 (28.8)	< 0.001
Septic shock	46 (7.9)	120 (17.3)	52 (22.3)	87 (28.4)	< 0.001
Distributive shock syndrome	74 (12.8)	53 (7.6)	21 (9.0)	28 (9.2)	0.018
Acute respiratory distress syndrome	20 (3.4)	44 (6.2)	15 (6.4)	24 (7.6)	0.032
Multiple organ failure	231 (36.1)	207 (27.1)	91 (34.7)	157 (47.6)	< 0.001
Postoperative procedures					
Percutaneous coronary intervention	8 (1.4)	20 (3.0)	7 (3.1)	13 (4.2)	0.084
Cardiac surgery	116 (19.6)	137 (19.2)	57 (24.3)	95 (30.3)	< 0.001
Abdominal surgery	18 (3.2)	37 (5.5)	7 (3.1)	23 (7.5)	0.015
Vascular surgery	56 (9.9)	80 (11.8)	34 (14.8)	38 (12.4)	0.250
In-hospital mortality	429 (66.1)	394 (50.8)	154 (58.6)	242 (72.7)	< 0.001
In-hospital mortality cause					< 0.001
Multiple organ failure	144 (34.9)	133 (36.8)	50 (35.0)	95 (42.6)	
Sepsis	15 (3.6)	30 (8.3)	17 (11.9)	23 (10.3)	
Persistent heart failure	153 (37.0)	120 (33.2)	62 (43.4)	80 (35.9)	
Distributive shock	15 (3.6)	5 (1.4)	1 (0.7)	1 (0.4)	
Bleeding	36 (8.7)	15 (4.2)	4 (2.8)	9 (4.0)	
Neurologic	23 (5.6)	29 (8.0)	1 (0.7)	4 (1.8)	
Bowel ischemia	5 (1.2)	11 (3.0)	4 (2.8)	2 (0.9)	
Other	22 (5.3)	18 (5.0)	4 (2.8)	9 (4.0)	

Data are reported as *n* (% as valid percentage excluding missing values) or median (interquartile range). *p* values by χ^2^ test (for categorical data) or Kruskal-Wallis test (for continuous data) indicate statistically significant differences between groups.

### Sensitivity Analysis

Sensitivity analysis revealed that 78% of the study population had their surgery between 2011 and 2020 (**Supplementary Tables 5–8** and **Supplementary Figs. 3** and **4**, http://links.lww.com/CCM/H558). There were only minor differences in preoperative and intraoperative characteristics. Distribution over ECMO duration strata was comparable, although slightly more patients underwent PC ECMO greater than 7 days in the 2011–2020 era compared with the 2000–2010 era (31.5 vs. 29.5%). Distribution of complications and in-hospital survival according to ECMO duration group in the 2011 to 2020 era was quite similar to the overall population.

### Subgroup Analysis

In the subgroup of patients with PC ECMO duration greater than 7 days (*n* = 596), survivors were younger, and less often had hypertension, diabetes mellitus, or chronic obstructive pulmonary disease (COPD) as comorbidities (**Supplementary Table 9**, http://links.lww.com/CCM/H558). Nonsurvivors more often required complex (> 3 procedures) surgeries with higher prevalence of aortic, mitral, or tricuspid valve surgery (**Supplementary Table 10**, http://links.lww.com/CCM/H558). PC ECMO management, LV unloading, timing of ECMO implantation, and cannulation approach did not differ between survivors and nonsurvivors (**Supplementary Table 11**, http://links.lww.com/CCM/H558). Complications such as bleeding, AKI, RV failure, septic shock, and multiple organ failure occurred more frequently in the nonsurvival group (**Supplementary Table 12**, http://links.lww.com/CCM/H558).

## DISCUSSION

The present study analyzed differences in characteristics and outcomes between patients who underwent different PC ECMO durations. With its size including over 2000 PC ECMO patients, and comprehensive documentation of ECMO-related complications and outcomes including long-term survival, this study can help better understand the relation between PC ECMO duration and outcomes. There were five main findings. First, prolonged PC ECMO support is applied often, with nearly 30% of PC ECMO runs lasting greater than 7 days. Second, the investigated preoperative and procedural characteristics showed no major differences according to ECMO duration groups. Third, postoperative complication rates (e.g., bleeding, AKI, arrhythmias, septic shock) are higher with longer PC ECMO duration. Fourth, results showed a U-shape curve for in-hospital mortality, with lowest mortality in patients undergoing 4–7 days of ECMO support. In the long PC ECMO duration group, nonsurvivors were of older age, had more comorbidities, and underwent more valvular and complex procedures. Finally, for those surviving ECMO weaning and hospital discharge, long-term survival was favorable regardless of PC ECMO duration.

Data from the PELS-1 study show that inability to be weaned from PC ECMO within 7 days is not a rare phenomenon, with nearly 30% of PC ECMO runs taking more than 7 days, and 16.5% of PC ECMO runs lasting more than 10 days. This is in line with Mariscalco et al (9), who reported 36% of PC ECMO runs lasting more than 7 days. In contrast, some older studies advocated early reevaluation at 48–72 hours or found no survivors beyond 8 days of PC ECMO support (21, 22). This points toward advances in the care for these critically ill patients and highlights how boundaries of PC ECMO have extended toward longer and more complex ECMO runs in a significant number of patients. Obviously, this increases demands, costs, and resources in terms of staff and intensive care capacity, which may be a complicating factor depending on available resources (23, 24).

We found only minor differences in preoperative and procedural characteristics in relation to PC ECMO duration. Patients undergoing prolonged PC ECMO tended to be a bit younger, and ejection fraction was 5% higher in the shortest PC ECMO duration group compared with other groups. However, we did not find, for example, preexisting low LVEF, preoperative shock, RV failure, or high European System for Cardiac Operative Risk Evaluation being associated with longer PC ECMO duration groups as clinical intuition may suggest. This is in line with results of earlier studies (9, 10). Furthermore, one could argue that different etiologies of PC cardiac failure (stunning, myocardial infarction, arrhythmias) may need different timeframes for cardiac recovery, but we did not find a relation between ECMO indication and ECMO duration.

Prolonged ECMO support in general is strongly associated with complications in the current and previous studies and different authors have noted the fine balance between the role of ECMO to support organ function and facilitate cardiac recovery vs. the ECMO-related risk of complications (7, 9, 10, 25). Bleeding is frequent in the PC ECMO population, due to previous cardiopulmonary bypass effects on the coagulation cascade, systemic anticoagulation, and ECMO-induced coagulation disorders, with up to 50% of patients in the longer ECMO duration groups needing rethoracotomy in our study, as observed also by Mariscalco et al (9). Nevertheless, caution is warranted in interpreting causality in relation to PC ECMO duration. Longer exposure to PC ECMO and the inherent need for anticoagulation and ECMO-induced coagulopathy may promote bleeding, but rethoracotomy may be a reason to postpone ECMO explantation as well, due to the time needed to overcome the new insult. The same may be true for arrhythmias, which we found to be associated with PC ECMO duration, but may as well be the reason for rather than the consequence of extra time on ECMO, to be sure to decannulate with a stable rhythm. Future detailed observational studies, documenting timing of occurrence of complication, and details of cardiac recovery can help resolve this issue. Finally, survival bias may also play a role in complication rates among PC ECMO duration groups.

Regarding mortality, overall rates follow a U-shape curve in relation to PC ECMO duration, with lowest mortality found in the 4–7 days group, which reflects earlier studies on both PC ECMO and venoarterial ECMO in general (7, 9). Uncontrolled bleeding and irreversible shock have previously been identified as contributors for the high mortality in short PC ECMO runs (7, 10). In the present study, bleeding was observed more often as cause of death in short PC ECMO group compared with the longer duration groups. For long PC ECMO runs, the above discussed association of ECMO duration with complications is paralleled with a decline in survival after 7 days of ECMO support. This may be the result of the complex interplay of unsuccessful cardiac recovery and additional organ injury due to complications. Indeed, multiple organ failure is the most prevalent cause of death in PC ECMO duration greater than 10 days, followed by persistent heart failure and sepsis. Timing of death is different as well between ECMO duration groups (Fig 1), with more in-hospital deaths occurring after decannulation in the longer ECMO duration groups. This so called “ECMO gap” has indeed been linked to complications in our cohort (18). Some authors have therefore advocated a reevaluation for usefulness of PC ECMO after 5–7 days of support, as chances for successful weaning drop (10, 26). The present study supports this, taking treatable complications like infection and sepsis into account and a structural evaluation of reasons for weaning failure (27). The PC ECMO duration greater than 7 days group showed that compared with survivors, nonsurvivors were older patients, who had more comorbidities like diabetes mellitus and COPD, and underwent more valvular and complex surgical procedures. Nonsurvivors also had more AKI, which likely contributed to lower chances of survival. All these factors together may help in the decision whether to prolong support after 7 days, which is complex. Therefore, multidisciplinary teams should take care of patients who undergo long PC ECMO runs, and preferably individual patient preferences should be taken into consideration by assessing those, at best before surgery (28).

There is scarce literature on the impact of ECMO duration on outcomes for other indications for venoarterial ECMO. An analysis of the Extracorporeal Life Support Organization registry showed a U-shape curve for mortality as well in a mixed cohort of venoarterial ECMO treated patients, and they could not identify an increase in mortality in ECMO durations beyond 12 days (7). Myocarditis and post-heart transplantation patients underwent longer ECMO durations than other categories, with better outcomes.

Although in the present study, in-hospital mortality was as high as 58.6% in patients with 7–10 days and 72.7% in those patients needing more than 10 days of PC ECMO support, the long-term prognosis of patients being discharged alive did not differ based on support duration. In other words, this important observation shows that patients who survive longer PC ECMO runs still have a good chance of survival after hospital discharge. Reevaluation after 7 days of support is justified given the decreasing chances for successful weaning, but the present results show that automatic PC ECMO withdrawal for futility is not indicated, and such an approach could even be harmful as perceived time restrictions may drive decision to stop in a way that becomes a self-fulfilling prophecy. However, identifying those with a reasonable chance for good outcome after greater than 7 days ECMO support remains a challenge.

Future studies should focus more in detail on the time course and interaction of complications, PC ECMO duration, and cardiac recovery. This may help individualize decision making regarding the usefulness of prolonged PC ECMO support vs. timely initiation of long-term therapies (LVAD, transplantation) for suitable candidates or palliative care for patients with no other options.

The retrospective nature of this study prevents any causal inferences. Furthermore, PC ECMO retrospective observational studies, by design, suffer from confounding by indication and survival bias may play a role in comparing subgroups of different PC ECMO duration. Although PELS is comprehensive, specific data on ECMO initiation criteria, protocols, weaning strategies, serial arterial lactate concentrations, longitudinal/serial data, vasopressor, and inotrope use are not captured by the database and could therefore not be included in this study. Similarly, exact timing of complications occurrence was not collected and reasons for withdrawing PC ECMO support may have varied according to participating hospital policies. The study period extended over 20 years guarantees a complete overview of PC ECMO on the one hand. On the other, differences in PC ECMO care over the study period might have changed. A sensitivity analysis was performed to control for these factors and outcomes were comparable. A partial overlapping with previously reported series cannot be excluded. In particular, we estimate an overlap with the study by Schaefer et al (29).

## CONCLUSIONS

In-hospital mortality after PC ECMO follows a U-shape, with lowest morality found after PC ECMO runs of 4–7 days. Nearly 30% of patients undergoing PC ECMO require support for more than 7 days. Longer PC ECMO support is associated with more complications like bleeding, AKI, septic shock, multiple organ failure, and increased mortality. This justifies critical reevaluation after ± 7 days of PC ECMO. Complications such as AKI, RV failure, and multiple organ failure should be considered in this reevaluation, as well as age, comorbidities, and valvular and complex surgery, as these characteristics were described more often in nonsurvivors among patients with longer PC ECMO runs (> 7 d). Future detailed observational studies should clarify the relationship between time on ECMO and complications, which may help to better identify those patients with a reasonable chance for hospital survival after longer PC ECMO runs. This is especially important since post-discharge survival is good despite PC ECMO support time.

## Supplementary Material
