# Supplementary material for: Characteristics and Outcomes of Prolonged Venoarterial Extracorporeal Membrane Oxygenation After Cardiac Surgery: The Post-Cardiotomy Extracorporeal Life Support (PELS-1) Cohort Study
Source: Crit Care Med. 2024 Jun 7;52(10):e490–502. doi: 10.1097/CCM.0000000000006349 (PMC11392071; doi:10.1097/CCM.0000000000006349)

Characteristics and Outcomes of Prolonged Extracorporeal Membrane Oxygenation after Cardiac Surgery:

the PELS-1 Multi-Centre Cohort Study

Corresponding author:

Jeroen J.H. Bunge

Thoraxcenter, Department of Cardiology,

Department of Intensive Care,

Erasmus MC,

Email: j.bunge@erasmusmc.nl

Phone:+31626966625

Table of contents:

List of PELS-1 Investigators 2

STROBE checklist 3

Supplementary Table 1: Variable and outcomes definitions. 5

Supplementary Table 2: Details of missing data for each variable of interest 8

Supplementary Table 3: Center characteristics. 10

Supplementary Table 4: Post-hoc analyses for significant between-group differences. 11

Supplementary Tables 5-8: Sensitivity analysis after excluding patients who received a post-cardiotomy extracorporeal membrane oxygenation before 2011 13

Supplementary Tables 9-12: Subgroup analysis: patients who remained on extracorporeal membrane oxygenation for more than 7 days 17

Supplementary Figure 1: Flow chart of study population 21

Supplementary Figure 2: Survival by ECMO day 22

Supplementary Figure 3: Survival by ECMO time, sensitivity analysis 2011-2020 23

Supplementary Figure 4: Kaplan-Meier curves, sensitivity analysis 2011-2020 24

List of PELS-1 Investigators

Samuel Heuts[1], Justine Ravaux[1], Anne-Kristin Schaefer[2], Luca Conci[2], Philipp Szalkiewicz[2], Jawad Khalil[3], Sven Lehmann[3], Jean-Francois Obadia[4], Nikolaos Kalampokas[5], Agne Jankuviene[6], Erwan Flecher[7], Kogulan Sriranjan[8], Michael A Mazzeffi[9], Nazli Vedadi[9], Marco Di Eusanio[10], Vitaly Sorokin[11], Ram Ramanathan[11], Alessandro Costetti[12], Chistof Schmid[13], Roberto Castillo[14], Vladimir Mikulenka[15], Tomas Grus[16], Marco Solinas[17]

1. Cardio-Thoracic Surgery Department, and Cardiovascular Research Institute Maastricht, Maastricht, The Netherlands.
2. Department of Cardiac Surgery, Medical University of Vienna, Vienna, Austria
3. Department of Cardiac Surgery, Leipzig Heart Center, Leipzig, Germany.
4. Department of Cardiac Surgery, Louis Pradel Cardiologic Hospital, Lyon, France
5. Department of Cardiac Surgery, Medical Faculty, Heinrich Heine University, Duesseldorf, Germany.
6. II Department of Anesthesiology, Centre of Anesthesia, Intensive Care and Pain management, Vilnius University Hospital Santariskiu Klinikos, Vilnius, Lithuania.
7. Division of Cardiothoracic and Vascular Surgery, Pontchaillou University Hospital, Rennes, France.
8. Department of Intensive Care Medicine, Center of Applied Medical Research, St Vincent's Hospital, Darlinghurst, NSW, Australia.
9. Departments of Medicine and Surgery, University of Maryland, Baltimore, USA.
10. SOD Cardiochirurgia Ospedali Riuniti 'Umberto I - Lancisi - Salesi' Università Politecnica delle Marche, Ancona, Italy.
11. Cardiothoracic Intensive Care Unit, National University Heart Centre, National University Hospital, Singapore, Singapore.
12. Cardiac Surgery Unit, Cardiac Thoracic and Vascular Department, Niguarda Hospital, Milan, Italy.
13. Department of Cardiothoracic Surgery, University Medical Center Regensburg, Regensburg, Germany.
14. ECMO Unit, Departamento de Anestesia, Clínica Las Condes, Las Condes, Santiago, Chile.
15. 2nd Department of Internal Medicine, Cardiovascular Medicine General Teaching Hospital and 1st Faculty of Medicine, Charles University in Prague, Prague, Czech Republic.
16. 2nd Department of Cardiovascular Surgery, First Faculty of Medicine, Charles University and General University Hospital in Prague, Prague, Czech Republic.
17. Ospedale del Cuore Fondazione Toscana "G. Monasterio", Massa, Italy.

STROBE Statement—Checklist of items that should be included in reports of ***cohort studies***

	Item No	Recommendation	Page No
**Title and abstract**	1	(*a*) Indicate the study's design with a commonly used term in the title or the abstract<br><br>(*b*) Provide in the abstract an informative and balanced summary of what was done and what was found	4
**Introduction**			
Background/rationale	2	Explain the scientific background and rationale for the investigation being reported	5
Objectives	3	State specific objectives, including any prespecified hypotheses	5
**Methods**			
Study design	4	Present key elements of study design early in the paper	6
Setting	5	Describe the setting, locations, and relevant dates, including periods of recruitment, exposure, follow-up, and data collection	6<br><br>S10
Participants	6	(*a*) Give the eligibility criteria, and the sources and methods of selection of participants. Describe methods of follow-up<br><br>(*b*) For matched studies, give matching criteria and number of exposed and unexposed	6
Variables	7	Clearly define all outcomes, exposures, predictors, potential confounders, and effect modifiers. Give diagnostic criteria, if applicable	6<br><br>S5
Data sources/ measurement	8*	For each variable of interest, give sources of data and details of methods of assessment (measurement). Describe comparability of assessment methods if there is more than one group	6
Bias	9	Describe any efforts to address potential sources of bias	11
Study size	10	Explain how the study size was arrived at	6
Quantitative variables	11	Explain how quantitative variables were handled in the analyses. If applicable, describe which groupings were chosen and why	7
Statistical methods	12	(*a*) Describe all statistical methods, including those used to control for confounding<br><br>(*b*) Describe any methods used to examine subgroups and interactions<br><br>(*c*) Explain how missing data were addressed<br><br>(*d*) If applicable, explain how loss to follow-up was addressed	7

		(*e*) Describe any sensitivity analyses	
**Results**			
Participants	13*	(a) Report numbers of individuals at each stage of study—eg numbers potentially eligible, examined for eligibility, confirmed eligible, included in the study, completing follow-up, and analysed	6<br>S22
		(b) Give reasons for non-participation at each stage	
		(c) Consider use of a flow diagram	
Descriptive data	14*	(a) Give characteristics of study participants (eg demographic, clinical, social) and information on exposures and potential confounders	7
		(b) Indicate number of participants with missing data for each variable of interest	S8
		(c) Summarise follow-up time (eg, average and total amount)	16
Outcome data	15*	Report numbers of outcome events or summary measures over time	8
Main results	16	(*a*) Give unadjusted estimates and, if applicable, confounder-adjusted estimates and their precision (eg, 95% confidence interval). Make clear which confounders were adjusted for and why they were included	7, 8
		(*b*) Report category boundaries when continuous variables were categorized	
		(*c*) If relevant, consider translating estimates of relative risk into absolute risk for a meaningful time period	
Other analyses	17	Report other analyses done—eg analyses of subgroups and interactions, and sensitivity analyses	7, 8
**Discussion**			
Key results	18	Summarise key results with reference to study objectives	9
Limitations	19	Discuss limitations of the study, taking into account sources of potential bias or imprecision. Discuss both direction and magnitude of any potential bias	11
Interpretation	20	Give a cautious overall interpretation of results considering objectives, limitations, multiplicity of analyses, results from similar studies, and other relevant evidence	9-11
Generalisability	21	Discuss the generalisability (external validity) of the study results	9
**Other information**			
Funding	22	Give the source of funding and the role of the funders for the present study and, if applicable, for the original study on which the present article is based	

*Give information separately for exposed and unexposed groups.

Supplementary Table 1 – Variable and outcomes definitions.

Variable	Definition
**Baseline characteristics**	
Hypertension	Systolic blood pressure >140mmHg or diastolic blood pressure >90mmHg, or use of antihypertensive agents to maintain normal blood pressure
Smoking	Active (smoking during the past 30 days) and more than 100 cigarettes during lifetime
COPD	Diagnosis of chronic obstructive pulmonary disease, any Gold classification
Peripheral arterial disease	Claudication, carotid occlusion or >50% stenosis, amputation for arterial disease or previous or planned intervention on the abdominal aorta, limb arteries or carotids
Pulmonary hypertension	Systolic pulmonary artery pressure >50mmHg
EuroSCORE II	European System for Cardiac Operative Risk Evaluation II proposing a risk assessment of cardiac surgical procedures which incorporates patient age, sex, diabetic status, pulmonary disease, neurological function, renal function, presence of active endocarditis, pre-operative state, procedural urgency and procedure type
NYHA class	Functional class of dyspnea according to the classification as proposed by the New York Heart Association
Preoperative cardiogenic shock	Preoperative state with life-threatening hypotension despite rapidly escalating inotropic support, critical organ hypoperfusion, with worsening acidosis and/or lactate levels
Preoperative cardiac arrest	Preoperative cardiopulmonary resuscitation in the 24 hours prior to surgery
Preoperative right ventricular failure	Evidence of right-sided structural and/or functional abnormalities in combination with clinical symptoms and signs of RV failure
Emergency surgery	Surgery before the beginning of the next working day after the decision to operate is made
Urgent surgery	Patients not electively admitted for operation but requiring surgery during the current admission without a possibility to be discharged before undergoing the definite procedure
Aortic vessel disease	And disease of the ascending aorta, aortic arch or proximal descending aorta warranting surgical correction during the current procedure
Aortic valve disease	Any aortic valve disease, including (prosthetic) aortic valve stenosis, regurgitation and endocarditis
Mitral valve disease	Any mitral valve disease, including (prosthetic) mitral valve stenosis, regurgitation and endocarditis

Tricuspid valve disease	Any tricuspid valve disease, including (prosthetic) tricuspid valve stenosis, regurgitation and endocarditis
Pulmonary valve disease	Any pulmonary valve disease, including (prosthetic) pulmonary valve stenosis, regurgitation and endocarditis
Active endocarditis	Patients still on antibiotic treatment for endocarditis at the time of surgery
Post LVAD right ventricular failure	RV failure as described previously in presence of LVAD
**Procedural characteristics**	
Ventricular surgery	Surgery performed to restore structural ventricular function, especially in case of ventricular aneurysm formation or rupture
Rhythm surgery	Surgical (either epicardial or endo-epicardial) ablation performed for atrial or ventricular arrhythmia
**Details on ECMO**	
Failure to wean	Failure to wean from CPB despite preload optimization and completeness of surgery
Arrhythmia	Refractory ventricular arrhythmia with uncontrollable hemodynamic consequences
Cardiac arrest	Abrupt loss of heart function despite acute and simple interventions such as pacing and defibrillation
Cardiogenic shock	State of life-threatening hypotension despite rapidly escalating inotropic support, critical organ hypoperfusion, with worsening acidosis and/or lactate levels
Right ventricular failure	Evidence of right-sided structural and/or functional abnormalities in combination with clinical symptoms and signs of RV failure
Respiratory failure	Reversible pulmonary disease which cannot anymore be managed by conventional mechanical ventilation, despite optimization of pharmacological interventions with or without prone positioning
Biventricular failure	Biventricular dysfunction accompanied by both signs and symptoms of right-sided and left-sided heart failure
Chest closed	Any cannulation condition in which the sternum is closed irrespective location of cannulas
Chest open	Any cannulation condition in which the sternum is left open irrespective of skin closure
**Postoperative outcomes**	

Stroke	Neurological dysfunction caused by focal brain or retinal ischemia with clinical symptoms lasting less more than 24 hours, with or without permanent disability
Arrhythmia	Any atrial or ventricular arrhythmia lasting more than 30 seconds
Leg ischemia	Clinical signs of lower extremity ischemia requiring intervention (either by vascular surgery or cannula removal)
Bowel ischemia	Intestinal ischemia with elevated lactate levels requiring abdominal surgical intervention
Acute kidney injury	Postoperative requirement for dialysis while not on dialysis before or duplication of preoperative creatinine levels (and absolute creatinine level >177µmol/L)
Pneumonia	Any (suspected) pulmonary infection treated with antibiotics
Septic shock	Sepsis with vasopressor requirement to maintain MAP >65mmHg and serum lactate levels greater than 2mmol/L in the absence of hypovolemia[43]
Distributive shock syndrome	MAP <50mmHg with cardiac index >2.5L/min/m^2, right atrial pressure <5mmHg, left atrial pressure <10mmHg an low systemic vascular resistance (<800 dyne/s/cm^{-5}) during intravenous norepinephrine infusion (>0.5µg/kg/min)[48]
ARDS	Acute diffuse inflammatory lung injury requiring invasive mechanical ventilation of extracorporeal membrane oxygenation
Multi-organ failure	Hypometabolic state with involvement of more than one organ as established by biochemical and/or radiological analysis

Supplementary Table 2 - Details of missing data for each variable of interest.

	**Overall population (n=2021)**	
Age	1	(0%)
Sex	1	(0%)
Body mass index	11	(0.5%)
Comorbidities		
Hypertension	1	(0%)
Diabetes mellitus	0	(0%)
Smoking	311	(15.4%)
COPD	84	(4.2%)
Dialysis	452	(22.4%)
Atrial fibrillation	1	(0%)
Peripheral artery disease	0	(0%)
Previous myocardial infarction	0	(0%)
Recent myocardial infarction (last 30 days)	71	(3.5%)
Previous PCI	17	(0.8%)
Previous stroke	0	(0%)
Previous TIA	226	(11.2%)
Pulmonary hypertension (>50 mmHg)	14	(0.7%)
Previous cardiac surgery	0	(0%)
Preoperative creatinine (umol/L)	139	(6.9%)
Euroscore II	583	(28.8%)
Preoperative cardiac status		
LVEF (%)	85	(4.2%)
NYHA class	98	(4.8%)
Euroscore II	583	(28.8%)
Cardiogenic shock	29	(1.4%)
Intubation	1	(0%)
Cardiac arrest	22	(1.1%)
Preoperative IABP	4	(0.2%)
Right ventricular failure	255	(12.6%)
Urgent surgery	20	(1.0%)
Emergency surgery	23	(1.1%)
Weight of surgery	0	(0%)
CABG	0	(0%)
Aortic valve surgery	0	(0%)
Mitral valve surgery	1	(0%)
Tricuspid valve surgery	0	(0%)
Aortic surgery	0	(0%)
Pulmonary valve surgery	0	(0%)
LVAD	0	(0%)
RVAD	0	(0%)
ASD repair	0	(0%)
VSD repair	0	(0%)
Ventricular surgery	0	(0%)
Rhythm surgery	0	(0%)
Pulmonary embolectomy	0	(0%)
Pulmonary endarterectomy	0	(0%)
Heart transplantation	0	(0%)
Off-pump surgery	27	(1.3%)
CPB time	172	(8.5%)
Cross clamp time	183	(9.1%)
Intraoperative lactate	1258	(62.2%)
PC ECMO indication	38	(1.9%)
Chest status	551	(27.3%)
Cannulation approach	0	(0%)
ECMO implant timing	0	(0%)
LV vent	356	(17.6%)
PC ECMO duration	0	(0%)

ICU stay	65	(3.2%)
Hospital stay	56	(2.8%)
Postoperative bleeding	27	(1.3%)
Requiring re-thoracotomy	125	(6.2%)
Cannulation site bleeding	29	(1.4%)
Diffuse no-surgical related bleeding	192	(9.5%)
Neurological complications		
Cerebral haemorrhage	108	(5.3%)
Stroke	13	(0.6%)
Arrhythmia	166	(8.2%)
Leg ischemia	110	(5.4%)
Cardiac arrest	166	(8.2%)
Bowel ischemia	165	(8.2%)
Right ventricular failure	205	(10.1%)
Acute kidney injury	172	(8.5%)
Pneumonia	206	(10.2%)
Septic shock	208	(10.3%)
Distributive shock syndrome	209	(10.3%)
ARDS	166	(8.2%)
MOF	26	(1.3%)
Postoperative procedures		
PCI	244	(12.1%)
Cardiac surgery	165	(8.2%)
Abdominal surgery	244	(12.1%)
Vascular surgery	240	(11.9%)
In-hospital mortality	0	(0%)

AMI, Acute Myocardial Infarction. ARDS, Acute Respiratory Distress Syndrome. ASD, Atrial Septal Defect. BMI, Body Mass Index. CABG, Coronary Artery Bypass Graft. COPD, Chronic Obstructive Pulmonary Disease. CPB, Cardiopulmonary Bypass. PC ECMO, post-cardiotomy extracorporeal membrane oxygenation. IABP, Intra-Aortic Balloon Pump. ICU, Intensive Care Unit. LV, Left Ventricular. LVEF, Left Ventricular Ejection Fraction. LVAD, Left Ventricular Assist Device. MOF, Multi-Organ Failure. NYHA, New York Heart Association. PCI, Percutaneous Coronary Intervention. RVAD, Right Ventricular Assist Device. TIA, Transient Ischemic Attack. VSD, Ventricular Septal Defect.

Supplementary Table 3 - Center characteristics.

	**Overall population (n=2021)**	**0-3 days (n=649)**	**4-7 days (n=776)**	**8-10 days (n=263)**	**>10 days (n=333)**	**p-value**
**Center type. n (%)**						<0.001
VAD center	97 (4.8%)	20 (1.0%)	34 (1.7%)	12 (0.6%)	31 (1.5%)	
HTx/VAD center	1754 (86.8%)	565 (28.0%)	672 (33.3%)	232 (11.5%)	285 (14.1%)	
non-HTx/non-VAD center	170 (8.4%)	64 (3.2%)	70 (3.5%)	19 (0.9%)	17 (0.8%)	
**Country. n (%)**						<0.001
Australia	71 (3.5%)	20 (3.1%)	37 (4.8%)	7 (2.7%)	7 (2.1%)	
Austria	487 (24.1%)	158 (24.3%)	210 (27.1%)	56 (21.3%)	63 (18.9%)	
Belgium	30 (1.5%)	11 (1.7%)	17 (2.2%)	1 (0.4%)	1 (0.3%)	
Chile	15 (0.7%)	2 (0.3%)	3 (0.4%)	1 (0.4%)	9 (2.7%)	
China	66 (3.3%)	24 (3.7%)	32 (4.1%)	5 (1.9%)	5 (1.5%)	
Colombia	40 (2.0%)	12 (1.8%)	12 (1.5%)	5 (1.9%)	11 (3.3%)	
Czech Republic	9 (0.4%)	3 (0.5%)	3 (0.4%)	0 (0.0%)	3 (0.9%)	
France	214 (10.6%)	88 (13.6%)	62 (8.0%)	37 (14.1%)	27 (8.1%)	
Germany	488 (24.1%)	161 (24.8%)	177 (22.8%)	82 (31.2%)	68 (20.4%)	
Italy	231 (11.4%)	51 (7.9%)	84 (10.8%)	30 (11.4%)	66 (19.8%)	
Lithuania	78 (3.9%)	17 (2.6%)	21 (2.7%)	11 (4.2%)	29 (8.7%)	
Netherlands	192 (9.5%)	66 (10.2%)	78 (10.1%)	20 (7.6%)	28 (8.4%)	
Singapore	28 (1.4%)	9 (1.4%)	13 (1.7%)	4 (1.5%)	2 (0.6%)	
South Korea	11 (0.5%)	6 (0.9%)	3 (0.4%)	0 (0.0%)	2 (0.6%)	
Thailand	21 (1.0%)	6 (0.9%)	10 (1.3%)	0 (0.0%)	5 (1.5%)	
USA	40 (2.0%)	15 (2.3%)	14 (1.8%)	4 (1.5%)	7 (2.1%)	

HTx, Heart Transplant. USA, United States of America. VAD, Ventricular Assist Device. P-values by chi squared indicate statistically significant differences between PC ECMO duration groups.

Supplementary Table 4 - Post-hoc analyses for significant between-group differences.

		**0-3 days (n=649) (A)**	**4-7 days (n=776) (B)**	**8-10 days (n=263) (C)**	**>10 days (n=333) (D)**
Age			D		B
Diabetes mellitus	No	C	C		
	Yes			A B	
Pulmonary hypertension (>50 mmHg)	No	B			
	Yes		A		
Left ventricular ejection fraction		D			A
Aortic valve surgery	No		C		
	Yes			B	
Mitral valve surgery	No	B			
	Yes		A		
Postoperative bleeding	No	B C D	D		
	Yes		A	A	A B
Postoperative bleeding requiring re-thoracotomy	No	C D	D		
	Yes			A	A B
Cannulation site bleeding	No	D			
	Yes				A
Arrhythmia	No	B C D	D		
	Yes		A	A	A B
Leg ischemia	No	D			
	Yes				A
Bowel ischemia	No	C			
	Yes			A	
Right ventricular failure	No	C	C		
	Yes			A B	
Acute kidney injury	No	B C D			
	Yes		A	A	A
Pneumonia	No	B C D			
	Yes		A	A	A
Septic shock	No	B C D	D		
	Yes		A	A	A B
Distributive shock syndrome	No		A		
	Yes	B			
Acute respiratory distress syndrome	No	D			
	Yes				A
Multi-organ failure	No	D	A D	D	
	Yes	B			A B C
Postoperative procedures: cardiac surgery	No	D	D		
	Yes				A B
Postoperative procedures: abdominal surgery	No	D			
	Yes				A
In-hospital mortality	No		A D	D	
	Yes	B			B C

In-hospital mortality cause	Multi-organ failure			
	Sepsis	A	A	A
	Persistent heart failure			
	Vasoplegia			
	Bleeding			
	Neurological	C D		
	Bowel ischemia			
	Other			

Results are based on two-sided tests. Significance level for upper case letters (A. B. C): 0.05. Tests are adjusted for all pairwise comparisons using the Bonferroni correction for multiple tests.

Sensitivity analysis after excluding patients who received a post-cardiotomy extracorporeal membrane oxygenation before 2011

Supplementary Table 5 - Baseline characteristics of the population 2011-2020 stratified according to post-cardiotomy extracorporeal membrane oxygenation (PC ECMO) implantation duration.

	**0-3 days (n=649)**		**4-7 days (n=776)**		**8-10 days (n=263)**		**>10 days (n=333)**		**p-value**
Demographics									
**Age (years)**	**65**	**(55-72)**	**66**	**(56-73)**	**64**	**(55-71)**	**63.5**	**(53.6-71)**	**0.062**
Female	189	(39.2%)	235	(39.3%)	82	(38.1%)	108	(38.6%)	0.989
**BMI (kg/m2)**	**26.2**	**(23.5-29.7)**	**25.9**	**(23.4-29.4)**	**27.3**	**(24.2-30.6)**	**26.8**	**(23.8-30.8)**	**0.025**
Comorbidities									
Hypertension	315	(69.1%)	376	(64.5%)	147	(73.5%)	178	(65.4%)	0.084
**Diabetes mellitus**	**117**	**(24.3%)**	**141**	**(23.5%)**	**68**	**(31.6%)**	**72**	**(25.7%)**	**0.12**
Smoking	104	(24.5%)	150	(28.4%)	49	(26.6%)	62	(24.8%)	0.534
COPD	44	(9.5%)	57	(10.0%)	22	(10.4%)	22	(8.2%)	0.828
Dialysis	38	(8.1%)	53	(9.0%)	22	(10.5%)	31	(11.3%)	0.487
Atrial fibrillation	122	(25.3%)	171	(28.5%)	56	(26.0%)	63	(22.6%)	0.284
Previous MI	111	(23.0%)	163	(27.2%)	58	(27.0%)	90	(32.1%)	0.054
Recent MI (<30 days)	47	(10.3%)	65	(11.1%)	21	(10.5%)	40	(14.7%)	0.297
Previous PCI	90	(18.8%)	96	(16.2%)	37	(17.4%)	51	(18.3%)	0.711
Previous stroke	71	(14.7%)	78	(13.0%)	35	(16.3%)	29	(10.4%)	0.21
Previous TIA	14	(3.1%)	13	(2.3%)	3	(1.5%)	3	(1.1%)	0.297
PHT (>50 mmHg)	72	(15.0%)	142	(23.8%)	47	(22.0%)	54	(19.5%)	0.004
Prior cardiac surgery	112	(23.2%)	159	(26.5%)	58	(27.0%)	74	(26.4%)	0.57
Creatinine (umol/L)	101	(80-138)	100	(80-136)	102	(79-133)	102	(80-146)	0.923
Preoperative Cardiac Status									
LVEF (%)	50	(35-60)	47	(30-60)	45	(30-58)	45	(30-55)	0.005
NYHA class									0.274
Class I	39	(8.5%)	44	(7.8%)	13	(6.3%)	22	(8.1%)	
Class II	95	(20.8%)	114	(20.2%)	42	(20.4%)	76	(27.8%)	
Class III	176	(38.5%)	240	(42.5%)	80	(38.8%)	96	(35.2%)	
Class IV	147	(32.2%)	167	(29.6%)	71	(34.5%)	79	(28.9%)	
Euroscore II	6.7	(2.7-18.3)	7.0	(3.1-17.3)	8.5	(2.6-18.3)	9.8	(3.7-20.0)	0.145
**Cardiogenic shock**	**98**	**(20.5%)**	**118**	**(20.0%)**	**55**	**(25.9%)**	**76**	**(27.2%)**	**0.042**
Intubation	57	(11.8%)	64	(10.7%)	28	(13.0%)	33	(11.8%)	0.819
Cardiac arrest	42	(8.8%)	38	(6.4%)	20	(9.4%)	21	(7.6%)	0.404
Preoperative IABP	29	(6.0%)	45	(7.5%)	19	(8.8%)	29	(10.4%)	0.164
RV failure	43	(10.1%)	49	(9.1%)	17	(8.9%)	27	(10.4%)	0.904
Urgent surgery	122	(25.7%)	125	(21.2%)	42	(19.6%)	53	(19.1%)	0.107
Emergency surgery	111	(23.4%)	143	(24.3%)	65	(30.4%)	77	(27.8%)	0.171

Data are reported as n (% as valid percentage excluding missing values). mean ± standard deviation or median (interquartile range). P-values by chi squared (for categorical data) or or Kruskal-Wallis test (for continuous data) indicate statistically significant differences between PC ECMO duration groups. Bold text indicates differences with the main analysis. BMI, Body Mass Index. COPD, Chronic Obstructive Pulmonary Disease. IABP,. Intra-Aortic Balloon Pump. LVEF, Left Ventricular Ejection Fraction. NYHA, New York Heart Association. PCI, Percutaneous Coronary Intervention. PHT, pulmonary hypertension. RV, right ventricular. TIA, Transient Ischemic Attack.

Supplementary Table 6 - Procedural characteristics stratified of patients 2011-2020 according to post-cardiotomy extracorporeal membrane oxygenation (PC ECMO) implantation duration.

	**0-3 days (n=649)**	**4-7 days (n=776)**	**8-10 days (n=263)**	**>10 days (n=333)**	**p-value**
Weight of surgery					0.206
Unknown	2 (0.4%)	3 (0.5%)	1 (0.5%)	3 (1.1%)	
Isolated CABG	78 (16.2%)	101 (16.9%)	45 (20.9%)	61 (21.8%)	
Isolated non-CABG	291 (60.4%)	338 (56.4%)	120 (55.8%)	141 (50.4%)	
2 procedures	26 (5.4%)	34 (5.7%)	18 (8.4%)	17 (6.1%)	
3 or more procedures	85 (17.6%)	123 (20.5%)	31 (14.4%)	58 (20.7%)	
Surgical Procedures					
CABG	209 (43.4%)	252 (42.1%)	107 (49.8%)	139 (49.6%)	0.072
**Aortic valve surgery**	**174 (36.1%)**	**198 (33.1%)**	**83 (38.6%)**	**98 (35.0%)**	**0.48**
Mitral valve surgery	130 (27.0%)	222 (37.1%)	58 (27.0%)	90 (32.3%)	0.002
Tricuspid valve surgery	57 (11.8%)	97 (16.2%)	31 (14.4%)	39 (13.9%)	0.24
Aortic surgery	116 (24.1%)	114 (19%)	39 (18.1%)	50 (17.9%)	0.09
Pulmonary valve surgery	5 (1.0%)	3 (0.5%)	2 (0.9%)	1 (0.4%)	0.621
LVAD	7 (1.5%)	6 (1.0%)	1 (0.5%)	6 (2.1%)	0.35
**RVAD**	**0 (0%)**	**1 (0.2%)**	**0 (0%)**	**3 (1.1%)**	**0.025**
ASD repair	8 (1.7%)	15 (2.5%)	1 (0.5%)	8 (2.9%)	0.202
VSD repair	13 (2.7%)	17 (2.8%)	9 (4.2%)	13 (4.6%)	0.382
Ventricular surgery	23 (4.8%)	23 (3.8%)	8 (3.7%)	12 (4.3%)	0.87
Rhythm surgery	14 (2.9%)	25 (4.2%)	3 (1.4%)	14 (5.0%)	0.117
Pulmonary embolectomy	2 (0.4%)	8 (1.3%)	3 (1.4%)	3 (1.1%)	0.447
**Pulmonary endarterectomy**	**2 (0.4%)**	**16 (2.7%)**	**3 (1.4%)**	**6 (2.1%)**	**0.036**
**Heart transplantation**	**45 (9.3%)**	**62 (10.4%)**	**14 (6.5%)**	**16 (5.7%)**	**0.081**
Procedural characteristics					
**Off-pump surgery**	**26 (5.5%)**	**23 (3.9%)**	**16 (7.4%)**	**7 (2.6%)**	**0.044**
Conversion to CPB	10 (38.5%)	7 (26.9%)	3 (18.8%)	2 (28.6%)	0.576
CPB time (min)	195 (133-288)	206 (149-289)	192 (141-262)	217 (130-297)	0.47
Cross clamp time (min)	98 (65-148)	107 (69-149)	102 (64-148)	103 (63-159)	0.637
Intraoperative lactate (mmol/L)	5.3 (3.0-10.0)	5.52 (2.6-8.6)	4.6 (2.4-6.9)	4.9 (2.7-7.8)	0.097

Data are reported as n (% as valid percentage excluding missing values) or median (interquartile range). P-values by chi squared (for categorical data) or or Kruskal-Wallis test (for continuous data) indicate statistically significant differences between groups. Bold text indicates differences with the main analysis. ASD, Atrial Septal Defect. CABG, Coronary Artery Bypass Graft. CPB, Cardiopulmonary Bypass. LVAD, Left Ventricular Assist Device. RVAD, Right Ventricular Assist Device. VSD, Ventricular Septal Defect.

Supplementary Table 7 - Details on post-cardiotmoy extracorporeal membrane oxygenation (PC ECMO) stratified according to PC ECMO duration. Patient years 2011-2020

	**0-3 days (n=649)**	**4-7 days (n=776)**	**8-10 days (n=263)**	**>10 days (n=333)**	**p-value**
**ECMO indication**					**0.02**
Failure to wean	177 (37.6%)	225 (38.0%)	68 (32.1%)	85 (31.5%)	
Acute pulmonary embolism	1 (0.2%)	1 (0.2%)	0 (0%)	1 (0.4%)	
Arrhythmia	10 (2.1%)	11 (1.9%)	7 (3.3%)	6 (2.2%)	
Cardiac arrest	63 (13.4%)	38 (6.4%)	15 (7.1%)	19 (7.0%)	
Cardiogenic shock	120 (25.5%)	157 (26.5%)	63 (29.7%)	84 (31.1%)	
Pulmonary haemorrhage	2 (0.4%)	4 (0.7%)	0 (0%)	0 (0%)	
Right ventricular failure	50 (10.6%)	77 (13.0%)	24 (11.3%)	43 (15.9%)	
Respiratory failure	10 (2.1%)	17 (2.9%)	12 (5.7%)	9 (3.3%)	
Biventricular failure	28 (5.9%)	53 (9.0%)	19 (9.0%)	21 (7.8%)	
Other	10 (2.1%)	9 (1.5%)	4 (1.9%)	2 (0.7%)	
Chest status					0.205
Chest closed	197 (60.4%)	226 (53.3%)	98 (60.1%)	114 (57.3%)	
Chest open	129 (39.6%)	198 (46.7%)	65 (39.9%)	85 (42.7%)	
Cannulation approach	0 (0%)	0 (0%)	0 (0%)	0 (0%)	0.195
Only central cannulation	72 (14.9%)	86 (14.4%)	29 (13.5%)	56 (20.0%)	
Only peripheral cannulation	231 (47.9%)	280 (46.7%)	97 (45.1%)	121 (43.2%)	
Mixed/switch cannulation	173 (35.9%)	221 (36.9%)	88 (40.9%)	102 (36.4%)	
Unknown	6 (1.2%)	12 (2.0%)	1 (0.5%)	1 (0.4%)	
**ECMO implant timing**					**0.007**
Intra-operative	297 (61.6%)	411 (68.6%)	130 (60.5%)	162 (57.9%)	
Post-operative	185 (38.4%)	188 (31.4%)	85 (39.5%)	118 (42.1%)	
LV vent	111 (27.5%)	147 (29.8%)	56 (33.3%)	89 (36.8%)	0.076

Data are reported as n (% as valid percentage excluding missing values). P-values by chi squared test indicate statistically significant differences between ECMO duration groups. Bold text indicates differences with the main analysis. ECMO, Extracorporeal Membrane Oxygenation. LV, Left Ventricular.

Supplementary Table 8 - Postoperative outcomes stratified according to post-cardiotomy extracorporeal membrane oxygenation (PC ECMO) duration. Patient years 2011-2020.

	**0-3 days (n=649)**	**4-7 days (n=776)**	**8-10 days (n=263)**	**>10 days (n=333)**	**p-value**
ICU stay (days)	3 (2-11)	14 (7-25)	20 (10-35)	22 (14-38)	<0.001
Hospital stay (days)	7 (2-24)	24 (11-44)	28.5 (11-47)	25 (17-46)	<0.001
Postoperative bleeding	220 (46.6%)	329 (56.0%)	131 (61.2%)	190 (68.6%)	<0.001
Requiring re-thoracotomy	139 (31.0%)	212 (38.0%)	93 (44.9%)	126 (47.7%)	<0.001
Cannulation site bleeding	43 (9.1%)	67 (11.4%)	30 (14.1%)	49 (17.8%)	0.004
Diffuse no-surgical related bleeding	94 (21.7%)	119 (22.1%)	53 (27.7%)	63 (24.8%)	0.32
Cerebral haemorrhage	11 (2.5%)	21 (3.7%)	6 (3.1%)	11 (4.1%)	0.619
Stroke	46 (9.6%)	64 (10.8%)	25 (11.7%)	36 (12.9%)	0.564
Arrhythmia	105 (24.0%)	189 (34.0%)	72 (37.3%)	108 (40.8%)	<0.001
Leg ischemia	33 (7.3%)	52 (9.1%)	16 (8.1%)	37 (13.9%)	0.028
Cardiac arrest	78 (17.8%)	81 (14.6%)	27 (14.0%)	38 (14.3%)	0.42
Bowel ischemia	14 (3.2%)	38 (6.8%)	16 (8.3%)	18 (6.8%)	0.029
Right ventricular failure	70 (16.4%)	93 (17.2%)	53 (27.9%)	64 (25.0%)	<0.001
Acute kidney injury	178 (40.8%)	313 (56.3%)	113 (58.9%)	175 (67.3%)	<0.001
Pneumonia	47 (11.1%)	139 (25.7%)	60 (31.4%)	70 (27.2%)	<0.001
Septic shock	29 (6.8%)	99 (18.4%)	44 (23.0%)	67 (26.1%)	<0.001
Distributive syndrome	62 (14.7%)	49 (9.1%)	20 (10.5%)	26 (10.2%)	0.048
ARDS	10 (2.3%)	28 (5.0%)	11 (5.7%)	18 (6.8%)	0.029
MOF	176 (36.7%)	157 (26.4%)	72 (33.5%)	125 (45.0%)	<0.001
Postoperative procedures					
PCI	8 (1.9%)	17 (3.2%)	7 (3.7%)	13 (5.0%)	0.147
Cardiac surgery	95 (21.7%)	121 (21.8%)	49 (25.4%)	88 (33.2%)	0.002
Abdominal surgery	11 (2.6%)	25 (4.7%)	7 (3.7%)	22 (8.5%)	0.004
Vascular surgery	42 (9.8%)	63 (11.9%)	30 (16%)	36 (13.9%)	0.136
In-hospital mortality	323 (67%)	298 (49.7%)	116 (54.0%)	202 (72.1%)	<0.001
In-hospital mortality cause	0 (0%)	0 (0%)	0 (0%)	0 (0%)	<0.001
Multi-organ failure	120 (38.6%)	98 (36.3%)	42 (39.6%)	79 (42.5%)	
Sepsis	8 (2.6%)	24 (8.9%)	10 (9.4%)	21 (11.3%)	
Persistent heart failure	110 (35.4%)	89 (33.0%)	43 (40.6%)	65 (34.9%)	
Distributive shock syndrome	13 (4.2%)	4 (1.5%)	1 (0.9%)	1 (0.5%)	
Bleeding	27 (8.7%)	10 (3.7%)	4 (3.8%)	8 (4.3%)	
Neurological	14 (4.5%)	23 (8.5%)	1 (0.9%)	4 (2.2%)	
Bowel ischemia	5 (1.6%)	10 (3.7%)	3 (2.8%)	1 (0.5%)	
Other	14 (4.5%)	12 (4.4%)	2 (1.9%)	7 (3.8%)	

Data are reported as n (% as valid percentage excluding missing values) or median (interquartile range). P-values by chi squared (for categorical data) or or Kruskal-Wallis test (for continuous data) indicate statistically significant differences between groups. Bold text indicates differences with the main analysis. ARDS, Acute Respiratory Distress Syndrome. ICU, intensive care unit. MOF, Multi-Organ Failure. PCI, Percutaneous Coronary Intervention.

Subgroup analysis: patients who remained on extracorporeal membrane oxygenation for more than 7 days

Supplementary Table 9 - Characteristics of patients who remained on post-cardiotomy extracorporeal membrane oxygenation (PC ECMO) for more than 7 days.

	PC ECMO > 7 days (n=596)	Survivors (n=200)	Non-survivors (n=396)	P-value
**Demographics**				
Age (years)	63 (54-71)	57 (49-66)	66 (58-72)	<0.001
Female	239 (40.1%)	82 (41.0%)	157 (39.6%)	0.791
BMI (kg/m2)	26.9 (23.9-30.6)	26.8 (23.6-30.6)	26.9 (24.1-30.7)	0.623
**Comorbidities**				
Hypertension	379 (66.5%)	111 (60.0%)	268 (69.6%)	0.029
Diabetes mellitus	173 (29.0%)	43 (21.5%)	130 (32.8%)	0.004
Smoking	136 (26.6%)	50 (30.1%)	86 (24.9%)	0.240
COPD	64 (11.1%)	14 (7.2%)	50 (13.2%)	0.035
Dialysis	61 (10.5%)	16 (8.2%)	45 (11.7%)	0.200
Atrial fibrillation	145 (24.4%)	37 (18.5%)	108 (27.3%)	0.020
Previous MI	169 (28.4%)	60 (30.0%)	109 (27.5%)	0.564
Recent MI (last 30 days)	69 (12.1%)	22 (11.9%)	47 (12.2%)	1.000
Previous PCI	99 (16.7%)	42 (21.1%)	57 (14.5%)	0.048
Previous stroke	78 (13.1%)	30 (15.0%)	48 (12.1%)	0.368
Previous TIA	8 (1.5%)	2 (1.1%)	6 (1.7%)	0.724
PHT (>50 mmHg)	130 (22.0%)	38 (19.0%)	92 (23.5%)	0.248
Prior cardiac surgery	161 (27.0%)	50 (25.0%)	111 (28.0%)	0.494
Creatinine (umol/L)	102 (79-141)	99 (79-126)	104 (79-149)	0.185
**Preoperative Cardiac Status**				
LVEF (%)	45 (30-58)	45 (28.5-60)	45 (30-57)	0.514
NYHA class				0.760
Class I	40 (6.9%)	15 (7.8%)	25 (6.5%)	
Class II	138 (23.9%)	44 (22.9%)	94 (24.4%)	
Class III	216 (37.4%)	68 (35.4%)	148 (38.4%)	
Class IV	183 (31.7%)	65 (33.9%)	118 (30.6%)	
Euroscore II	9.0 (2.9-19.5)	8.8 (2.5-18.3)	9.0 (3.2-20.8)	0.373
Cardiogenic shock	144 (24.4%)	50 (25.5%)	94 (23.8%)	0.684
Intubation	72 (12.1%)	26 (13.0%)	46 (11.6%)	0.690
Cardiac arrest	52 (8.8%)	17 (8.7%)	35 (8.9%)	1.000
Preoperative IABP	63 (10.6%)	21 (10.5%)	42 (10.6%)	1.000
RV failure	54 (10.3%)	16 (9.2%)	38 (10.8%)	0.649
Urgent surgery	115 (19.4%)	36 (18.2%)	79 (20.0%)	0.660
Emergency surgery	162 (27.4%)	59 (29.9%)	103 (26.1%)	0.329

Data are reported as n (% as valid percentage excluding missing values) or median (interquartile range). P-values by chi squared (for categorical data) or or Kruskal-Wallis test (for continuous data) indicate statistically significant differences between groups. BMI, Body Mass Index. COPD, Chronic Obstructive Pulmonary Disease. IABP, Intra-Aortic Balloon Pump. LVEF, Left Ventricular Ejection Fraction. MI, myocardial infarction. NYHA, New York Heart Association. PCI, Percutaneous Coronary Intervention. PHT, pulmonary hypertension. RV, right ventricular. TIA, Transient Ischemic Attack.

Supplementary Table 10 - Procedural characteristics of patients who remained on post-cardiotomy extracorporeal membrane oxygenation (PC ECMO) for more than 7 days.

	**ECMO > 7 days (n=596)**		**Survivors (n=200)**		**Non-survivors (n=396)**		**p-value**
**Weight of surgery**							0.012
Unknown	5	(0.8%)	3	(1.5%)	2	(0.5%)	
Isolated CABG	119	(20.0%)	50	(25.0%)	69	(17.4%)	
Isolated non-CABG	318	(53.4%)	104	(52.0%)	214	(54.0%)	
2 procedures	46	(7.7%)	19	(9.5%)	27	(6.8%)	
3 or more procedures	108	(18.1%)	24	(12.0%)	84	(21.2%)	
**Surgical procedures**							
CABG	279	(46.8%)	100	(50.0%)	179	(45.2%)	0.297
Aortic valve surgery	220	(36.9%)	57	(28.5%)	163	(41.2%)	0.003
Mitral valve surgery	183	(30.8%)	51	(25.6%)	132	(33.3%)	0.06
Tricuspid valve surgery	81	(13.6%)	16	(8.0%)	65	(16.4%)	0.005
Aortic surgery	100	(16.8%)	31	(15.5%)	69	(17.4%)	0.643
Pulmonary valve surgery	3	(0.5%)	2	(1.0%)	1	(0.3%)	0.262
LVAD	7	(1.2%)	2	(1.0%)	5	(1.3%)	1
RVAD	4	(0.7%)	2	(1.0%)	2	(0.5%)	0.605
ASD repair	12	(2.0%)	3	(1.5%)	9	(2.3%)	0.759
VSD repair	27	(4.5%)	9	(4.5%)	18	(4.5%)	1
Ventricular surgery	22	(3.7%)	7	(3.5%)	15	(3.8%)	1
Rhythm surgery	19	(3.2%)	6	(3.0%)	13	(3.3%)	1
Pulmonary embolectomy	8	(1.3%)	4	(2.0%)	4	(1.0%)	0.452
Pulmonary endarterectomy	19	(3.2%)	5	(2.5%)	14	(3.5%)	0.625
Heart transplantation	45	(7.6%)	18	(9.0%)	27	(6.8%)	0.412
**Procedural characteristics**							
Off-pump surgery	24	(4.1%)	13	(6.6%)	11	(2.8%)	0.044
Conversion to CPB	5	(20.8%)	1	(7.7%)	4	(36.4%)	0.142
CPB time (min)	210	(142-290)	202	(132-291)	218	(146-290)	0.263
Cross clamp time (min)	103	(65-157)	94	(63-142)	110	(67-161)	0.093

Data are reported as n (% as valid percentage excluding missing values) or median (interquartile range). P-values by chi squared (for categorical data) or or Kruskal-Wallis test (for continuous data) indicate statistically significant differences between groups. ASD, Atrial Septal Defect. CABG, Coronary Artery Bypass Graft. CPB, Cardiopulmonary Bypass. LVAD, Left Ventricular Assist Device. RVAD, Right Ventricular Assist Device. VSD, Ventricular Septal Defect.

Supplementary Table 11 - Details on post-cardiotomy extracorporeal membrane oxygenation (PC ECMO) of patients who remained on support for more than 7 days.

	PC ECMO > 7 days (n=596)		Survivors (n=200)		Non-survivors (n=396)		P-value
**ECMO indication**							0.05
Failure to wean	206	(35.3%)	66	(33.7%)	140	(36.2%)	
Acute pulmonary embolism	1	(0.2%)	0	(0%)	1	(0.3%)	
Arrhythmia	13	(2.2%)	8	(4.1%)	5	(1.3%)	
Cardiac arrest	43	(7.4%)	13	(6.6%)	30	(7.8%)	
Cardiogenic shock	165	(28.3%)	59	(30.1%)	106	(27.4%)	
Pulmonary haemorrhage	1	(0.2%)	1	(0.5%)	0	(0%)	
Right ventricular failure	78	(13.4%)	26	(13.3%)	52	(13.4%)	
Respiratory failure	26	(4.5%)	10	(5.1%)	16	(4.1%)	
Biventricular failure	43	(7.4%)	8	(4.1%)	35	(9%)	
Other	7	(1.2%)	5	(2.6%)	2	(0.5%)	
**Cannulation approach**							0.323
Only central cannulation	107	(18.0%)	28	(14.0%)	79	(19.9%)	
Only peripheral cannulation	269	(45.1%)	96	(48.0%)	173	(43.7%)	
Mixed/switch cannulation	218	(36.6%)	75	(37.5%)	143	(36.1%)	
Unknown	2	(0.3%)	1	(0.5%)	1	(0.3%)	
**ECMO implant timing**							
Intra-operative	353	(59.2%)	119	(59.5%)	234	(59.1%)	
Post-operative	243	(40.8%)	81	(40.5%)	162	(40.9%)	
LV vent	175	(35.3%)	57	(34.5%)	118	(35.6%)	0.84

Data are reported as n (% as valid percentage excluding missing values). ECMO, Extracorporeal Membrane Oxygenation. LV, Left Ventricular. P-values by chi squared test indicate statistically significant differences between groups.

Supplementary Table 12 - Postoperative outcomes of patients who remained on post-cardiotomy extracorporeal membrane oxygenation (PC ECMO) for more than 7 days.

	**ECMO > 7 days (n=596)**	**Survivors (n=200)**	**Non-survivors (n=396)**	**P-value**
ICU stay (days)	21 (13-37)	34 (22-49)	15 (11-26)	0.002
Hospital stay (days)	25 (14-46)	49 (33-80)	18 (12-29)	0.032
Postoperative bleeding	395 (66.7%)	118 (59.6%)	277 (70.3%)	0.01
Requiring re-thoracotomy	275 (48.5%)	84 (44.7%)	191 (50.4%)	0.212
Cannulation site bleeding	95 (16.1%)	24 (12.2%)	71 (18.1%)	0.075
Diffuse no-surgical related bleeding	142 (26.4%)	40 (22.7%)	102 (28.2%)	0.211
Cerebral haemorrhage	19 (3.4%)	6 (3.3%)	13 (3.4%)	1
Stroke	73 (12.3%)	32 (16.1%)	41 (10.4%)	0.048
Arrhythmia	225 (41%)	78 (43.8%)	147 (39.6%)	0.355
Leg ischemia	67 (11.9%)	14 (7.7%)	53 (14.0%)	0.037
Cardiac arrest	82 (14.9%)	15 (8.4%)	67 (18.1%)	0.003
Bowel ischemia	43 (7.8%)	9 (5.1%)	34 (9.1%)	0.126
Right ventricular failure	141 (26.3%)	24 (13.8%)	117 (32.2%)	<0.001
Heart transplant	38 (8.1%)	22 (14.1%)	16 (5.1%)	0.002
Acute kidney injury	358 (65.8%)	99 (56.6%)	259 (70.2%)	0.002
Pneumonia	158 (29.3%)	60 (34.3%)	98 (26.9%)	0.086
Septic shock	139 (25.8%)	31 (17.7%)	108 (29.7%)	0.003
Distributive shock syndrome	49 (9.1%)	9 (5.2%)	40 (11.0%)	0.036
ARDS	39 (7.1%)	10 (5.6%)	29 (7.8%)	0.381
MOF	248 (41.9%)	21 (10.5%)	227 (57.9%)	<0.001
Postoperative procedures				
PCI	20 (3.7%)	8 (4.6%)	12 (3.3%)	0.475
Cardiac surgery	152 (27.7%)	49 (27.5%)	103 (27.8%)	1
Abdominal surgery	30 (5.6%)	10 (5.7%)	20 (5.6%)	1
Vascular surgery	72 (13.4%)	18 (10.3%)	54 (15.0%)	0.176

Data are reported as n (% as valid percentage excluding missing values) or median (interquartile range). P-values <0.05 by chi squared (for categorical data) or or Kruskal-Wallis test (for continuous data) indicate statistically significant differences between groups. ARDS, Acute Respiratory Distress Syndrome. ICU, intensive care unit. MOF, Multi-Organ Failure. PCI, Percutaneous Coronary Intervention. TIA, Transient Ischemic Attack.

Supplementary Figures

Supplementary Figure 1: Flow chart of study population

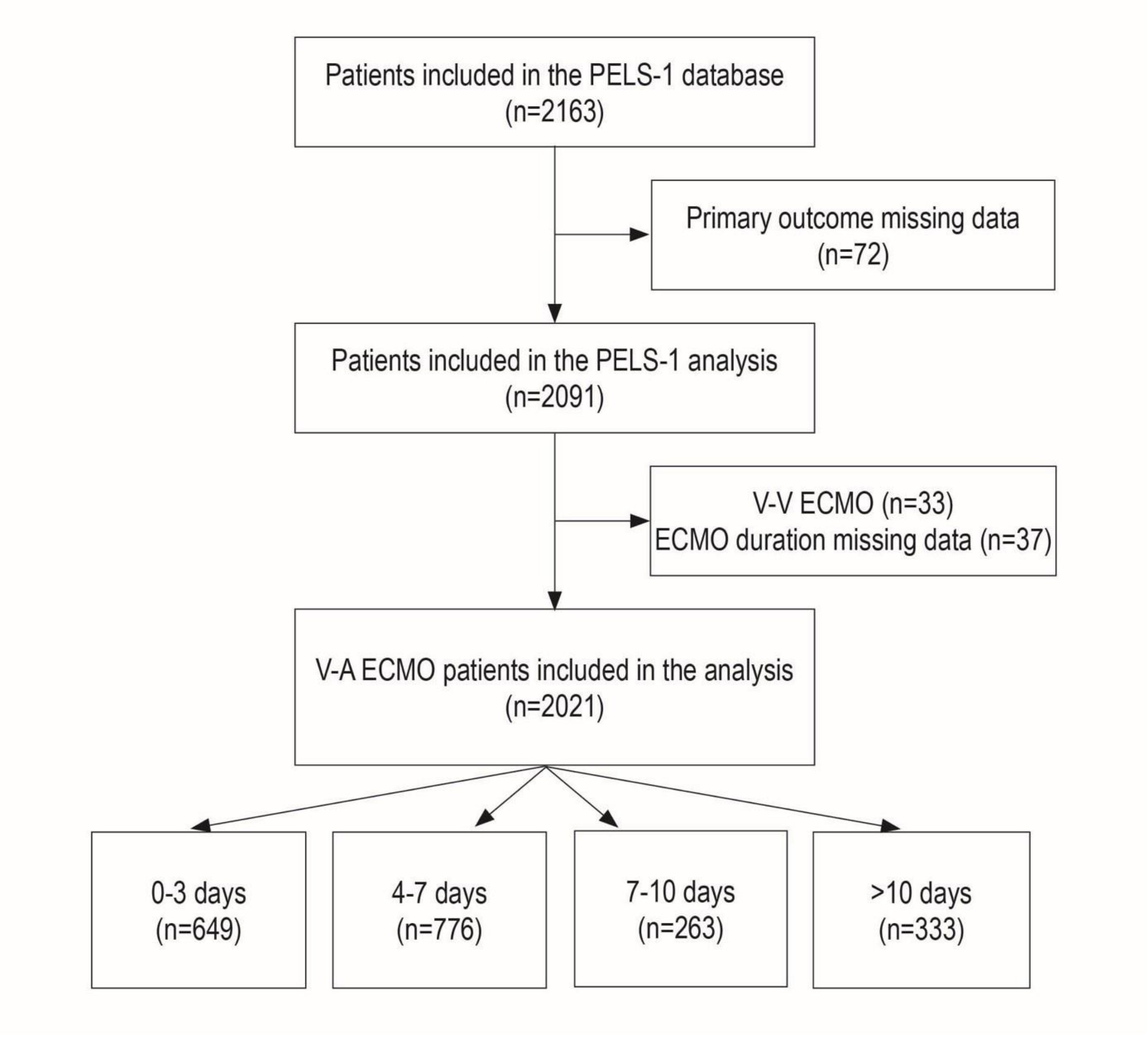

Supplementary Figure 2 – Stacked bar plots representing mortality while on post-cardiotomy extracorporeal membrane oxygenation (PC ECMO). in hospital mortality after ECMO explantation. and hospital survival by days of support.

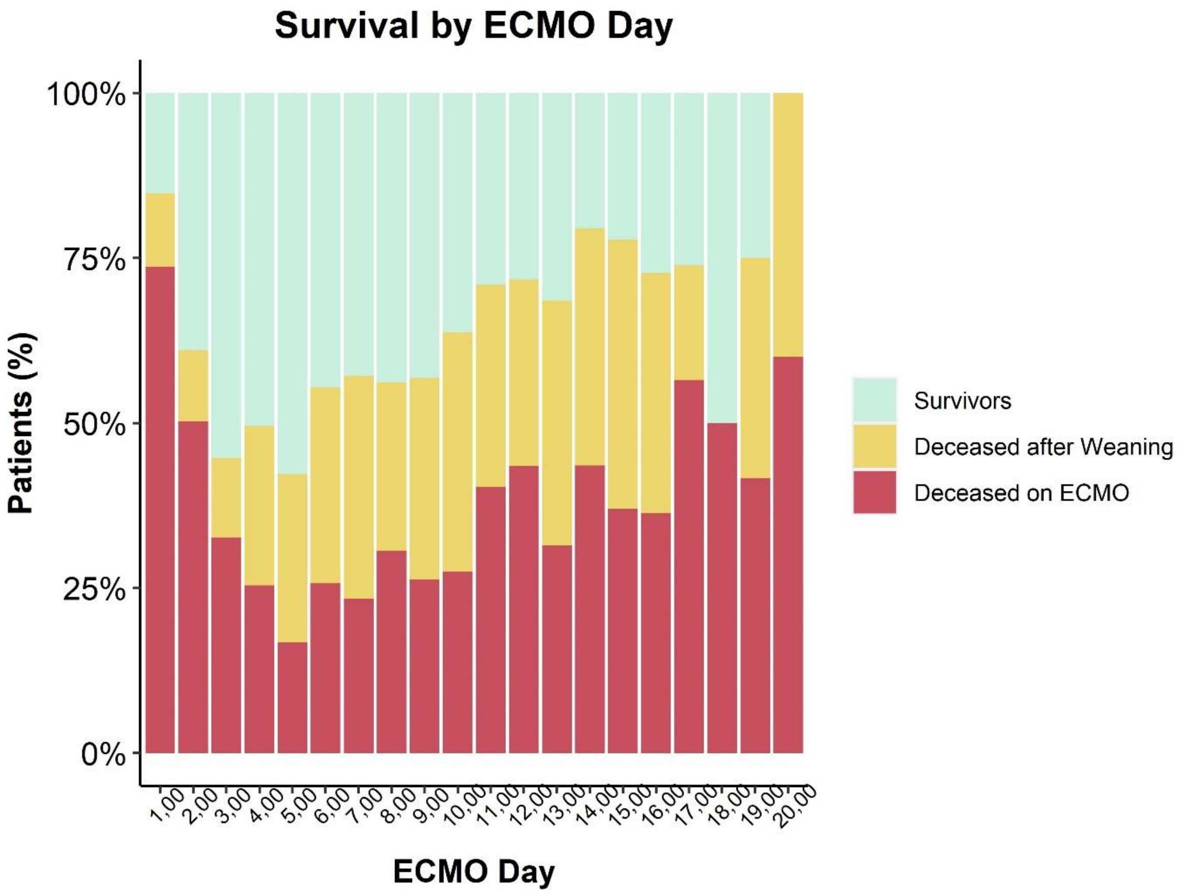

Supplementary Figure 3: Stacked bar plots representing mortality while on post-cardiotomy extracorporeal membrane oxygenation (PC ECMO). in hospital mortality after PC ECMO explantation. and hospital survival by PC ECMO duration groups. excluding patients who received a post-cardiotomy extracorporeal membrane oxygenation before 2011 (Sensitivity analysis).

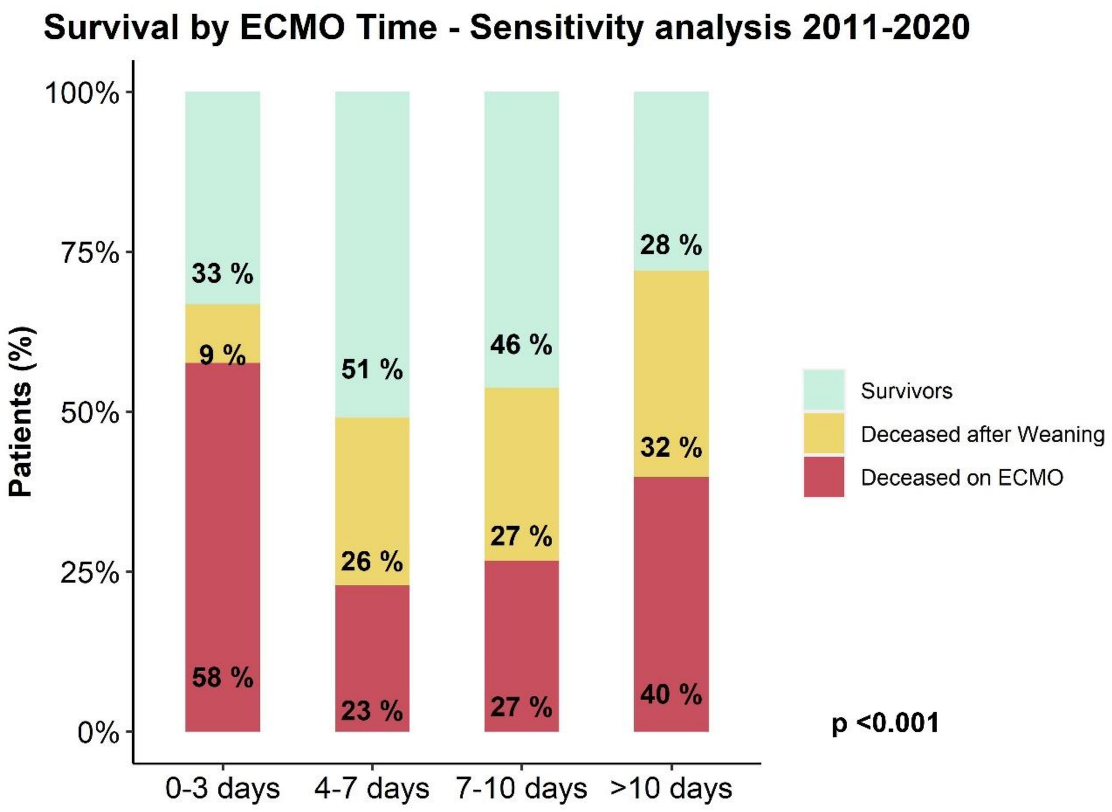

Supplementary Figure 4 – Kaplan-Meier curves with 95% confidence intervals excluding patients who received a post-cardiotomy extracorporeal membrane oxygenation before 2011 (Sensitivity analysis).

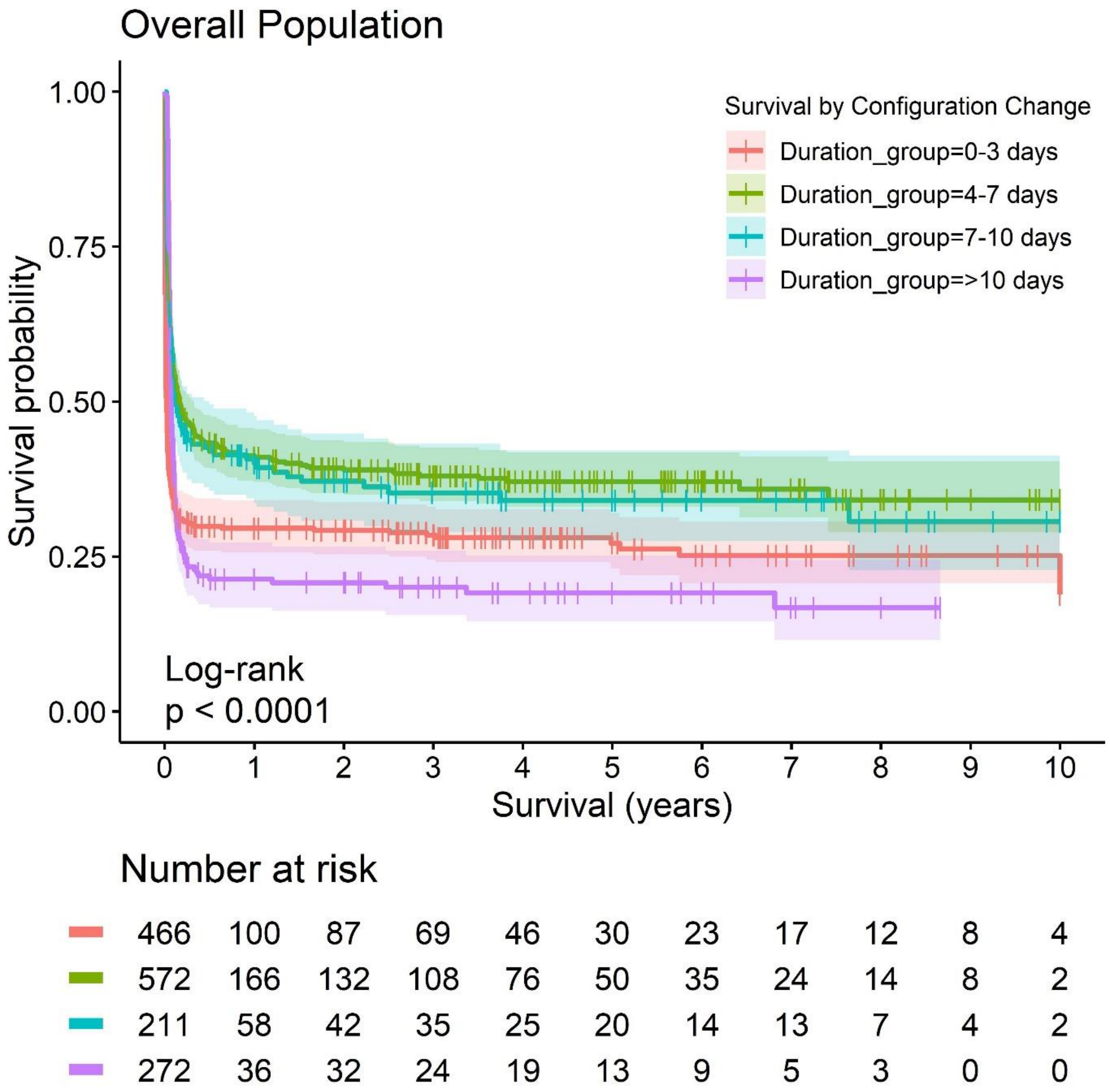

Supplement: Supplementary file 1 [file ccm-52-e490-s001.pdf]
